# Serum miRNA-based diagnostic models for endometriosis: from discovery to validation

**DOI:** 10.1093/humrep/deaf221

**Published:** 2025-11-21

**Authors:** Antonella Ravaggi, Cosetta Bergamaschi, Jacopo Conforti, Giuseppe Ciravolo, Laura Zanotti, Aline S C Fabricio, Massimo Gion, Elia Cappelletto, Antonette E Leon, Diego Oreste Rossetti, Cesare Romagnolo, Stefano Calza, Eliana Bignotti, Franco Odicino

**Affiliations:** Department of Clinical and Experimental Sciences, University of Brescia, Brescia, Italy; Department of Obstetrics and Gynecology, ASST Spedali Civili di Brescia, Brescia, Italy; Angelo Nocivelli Institute of Molecular Medicine, ASST Spedali Civili di Brescia, University of Brescia, Brescia, Italy; Department of Obstetrics and Gynecology, ASST Spedali Civili di Brescia, Brescia, Italy; Angelo Nocivelli Institute of Molecular Medicine, ASST Spedali Civili di Brescia, University of Brescia, Brescia, Italy; Residency Program for Clinical Pathology and Clinical Biochemistry, University of Brescia, Brescia, Italy; Department of Clinical and Experimental Sciences, University of Brescia, Brescia, Italy; Department of Obstetrics and Gynecology, ASST Spedali Civili di Brescia, Brescia, Italy; Clinical Trial Center, Translational Research and Phase I Unit, ASST Spedali Civili di Brescia, Brescia, Italy; Basic and Translational Oncology, Veneto Institute of Oncology IOV-IRCCS, Padova, Italy; Department of Clinical Pathology, Regional Center for Biomarkers, AULSS3 Serenissima, Venezia, Italy; Basic and Translational Oncology, Veneto Institute of Oncology IOV-IRCCS, Padova, Italy; Department of Clinical Pathology, Regional Center for Biomarkers, AULSS3 Serenissima, Venezia, Italy; Department of Obstetrics and Gynecology, ASST Spedali Civili di Brescia, Brescia, Italy; Unit of Obstetrics and Gynecology, Dell’Angelo Hospital, Mestre (VE), Italy; Unit of Biostatistics and Bioinformatics, Department of Molecular and Translational Medicine, University of Brescia, Brescia, Italy; Department of Obstetrics and Gynecology, ASST Spedali Civili di Brescia, Brescia, Italy; Angelo Nocivelli Institute of Molecular Medicine, ASST Spedali Civili di Brescia, University of Brescia, Brescia, Italy; Department of Clinical and Experimental Sciences, University of Brescia, Brescia, Italy; Department of Obstetrics and Gynecology, ASST Spedali Civili di Brescia, Brescia, Italy

**Keywords:** miRNA, serum, diagnosis, machine learning, endometriosis

## Abstract

**STUDY QUESTION:**

Can a serum miRNA signature serve as a potential diagnostic biomarker for endometriosis (END)?

**SUMMARY ANSWER:**

A miRNA-based diagnostic model demonstrated an accuracy of 65.8% in distinguishing END patients from control subjects (CTR), demonstrating good sensitivity but limited specificity.

**WHAT IS KNOWN ALREADY:**

Existing research has examined the potential utility of circulating miRNAs as biomarkers for END diagnosis, revealing their differential expression between women with END and CTR. Nevertheless, the findings remain conflicting, and at present, neither a single miRNA nor a panel of them has yet been established as a reliable diagnostic test in clinical practice for the management of END.

**STUDY DESIGN, SIZE, DURATION:**

We previously reported different miRNA expression patterns in serum samples from 67 END patients and 60 CTR by high-throughput RT-qPCR. In this multicenter study, a total of 364 patients with pathology-confirmed diagnosis of END or a benign non-END gynecological condition were retrospectively selected from a biobank or prospectively enrolled. The aims of the present study were to analyze, in the entire cohort of patients, a set of 23 potential diagnostic miRNAs via RT-qPCR and to create models capable of diagnosing END through cross-validated machine learning algorithms.

**PARTICIPANTS/MATERIALS, SETTING, METHODS:**

Total RNA was extracted from serum samples collected before surgical treatment and miRNAs were evaluated by RT-qPCR. Diagnostic models were developed using both the Random Forest and Logistic Regression algorithms. The performance assessment of the various models was derived from internal validation, using repeated cross-validation.

**MAIN RESULTS AND THE ROLE OF CHANCE:**

The most effective diagnostic model was constructed with 11 miRNAs: miR-140-3p, miR-181a-5p, miR-192-5p, miR-22-3p, miR-29a-3p, miR-30b-5p, miR-338-3p, miR-340-5p, miR-342-3p, miR-486-5p, and miR-652-3p. The diagnostic efficacy of the model was defined by an AUC of 70.4%, a sensitivity of 75.6%, a specificity of 53.5%, and an accuracy of 65.8%. The model that used six miRNAs (miR-192-5p, miR-30b-5p, miR-335-5p, miR-338-3p, miR-486-5p, miR-652-3p) was the best at identifying deep infiltrating endometriosis compared to the control group, with an AUC of 80.4% and an accuracy of 75.9%. A lower accuracy was achieved by the model differentiating ovarian endometrioma (OMA) from CTR (AUC = 65.8%; accuracy = 62.4%).

**LARGE SCALE DATA:**

miRNA expression profiles have been deposited in NCBI’s Gene Expression Omnibus and are accessible through GEO Series accession numbers GSE279435.

**LIMITATIONS, REASONS FOR CAUTION:**

Despite the internal cross-validation, the models still need to be tested on larger cohorts of prospectively enrolled patients across several centers to enhance their accuracy and robustness. This testing will also facilitate monitoring the model in a real-world setting, potentially integrating the miRNA-based model with other diagnostic tools, such as ultrasound.

**WIDER IMPLICATIONS OF THE FINDINGS:**

If proven effective in larger cohorts, this model could serve as a tool for the diagnosis of END, thereby enhancing early identification and clinical care of this disease. Moreover, given its low false negative rate, the miRNA-based model may be useful as a screening tool to help identify patients who are likely to have END but warrant further evaluation to confirm END diagnosis.

**STUDY FUNDING/COMPETING INTEREST(S):**

This research was financed by the Italian Ministry of Health, grant number “LOMBARDIA ENDO-2021-12371946”, project title: FREEDOM TRIAL, and the Italian Ministry of Research (PRIN project n. 2022SYXEHJ to SC). The authors disclose no conflicts of interest.

**TRIAL REGISTRATION NUMBER:**

N/A.

## Introduction

Endometriosis (referred to herein as END) is a persistent, benign inflammation impacting the female reproductive system and pelvic peritoneum that is caused by the aberrant localization of endometrial cells in various organs beyond the uterine lining (Giudice and Kao, 2004; Pascoal *et al.*, 2022). END affects ∼5–10% of women of reproductive age and is detected in up to 50% of women with infertility (Taylor *et al.*, 2021). This illness manifests in three primary forms, which are superficial peritoneal lesions, ovarian endometrioma (OMA), and deep infiltrating endometriosis (DIE) (Chapron *et al.*, 2019). The predominant sites include the ovaries, peritoneum, rectum, rectosigmoid region, rectovaginal pouch, and uterosacral ligaments. Occasionally, END may also affect organs outside the female genital system (Lee *et al.*, 2021; Utkarsh *et al.*, 2023). Symptoms may differ significantly in nature and intensity, and in certain instances, patients can be completely asymptomatic. This different disease presentation frequently results in misdiagnosis or delayed recognition of the disorder, causing physical, mental, and social distress in affected women (Ballard *et al.*, 2006). Transvaginal ultrasound is the first imaging technique for diagnosing END, while MRI serves as an adjunctive tool, mainly to investigate END lesions above the pelvis. In some cases, laparoscopic surgery and histological verification may be necessary for a conclusive diagnosis. Nonetheless, because of its invasive nature, laparoscopy is frequently deferred in women of reproductive age, leading to a postponement of diagnosis and treatment (Ahn *et al.*, 2017; Rolla, 2019). The identification of non-invasive biomarkers for END diagnosis would lead to substantial progress in its management (Agrawal *et al.*, 2018). Specific circulating markers can facilitate the detection of END using liquid biopsy, providing a less intrusive alternative to conventional tissue biopsies (Agrawal *et al.*, 2018; Nikanjam *et al.*, 2022). In addition, non-invasive diagnostic techniques could facilitate the monitoring of disease development and treatment efficacy, enhancing patient comfort and decreasing healthcare costs (Agrawal *et al.*, 2018).

Micro-RNAs (miRNAs) are single-stranded non-coding RNAs, ∼22 nucleotides long, that regulate gene expression by inhibiting translation or promoting the degradation of messenger RNA transcripts (Cho *et al.*, 2015; Bagheri *et al.*, 2024; Chico-Sordo *et al.*, 2024). MiRNAs are found both inside cells and in circulation, demonstrating significant stability (Bjorkman and Taylor, 2019; Bagheri *et al.*, 2024). These molecules govern numerous biological processes, encompassing endometrial functions and the progression of gynecological disorders such as END (Ohlsson Teague *et al.*, 2010; Chico-Sordo *et al.*, 2024). Given their presence in biofluids and potential role in the pathophysiology of END, miRNAs are promising candidates for non-invasive biomarkers in END diagnosis, as evidenced in multiple studies (Wang *et al.*, 2013, 2016, 2018; Cosar *et al.*, 2016; Pateisky *et al.*, 2018; Nisenblat *et al.*, 2019; Vanhie *et al.*, 2019; Moustafa *et al.*, 2020; Papari *et al.*, 2020; Misir *et al.*, 2021; Bendifallah *et al.*, 2022a,b; Lin *et al.*, 2023; Walasik *et al.*, 2023; Chico-Sordo *et al.*, 2024). Currently, no single miRNA or panel of miRNAs has been identified and validated as a reliable blood test for the diagnosis of END in clinical applications.

In our previous study (Ravaggi *et al.*, 2024), 16 miRNAs differentially expressed between the END and CTR groups were identified through liquid biopsy using OpenArray technology. Based on these findings, in the current study, we aimed to validate a selected subset of miRNAs using RT-qPCR in samples from a larger cohort, which were both retrospectively selected from two biobanks and prospectively collected from patients enrolled in the Division of Obstetrics and Gynaecology of two different Italian hospitals. The aim of this study was to build diagnostic models discriminating all END or END subgroups (OMA, DIE) from non-endometriotic gynecological benign lesions.

## Materials and methods

### Patients and biological samples

In this multicenter study, two sub-cohorts of patients were analyzed. The first cohort included 125 END patients and 120 CTR patients with a benign non-endometriotic gynecological condition, whose serum samples were stored in the biorepositories of “A. Nocivelli” Institute of Molecular Medicine (“ASST-Spedali Civili di Brescia,” Italy) and CRIBT (“Azienda ULSS 3 Serenissima,” Italy). These patients retrospectively selected from biobank databases had previously been diagnosed and treated in the Divisions of Obstetrics and Gynaecology of “ASST Spedali Civili di Brescia” and “Azienda ULSS3 Serenissima” between January 2009 and February 2012.

The second sub-cohort included patients with suspected benign gynecological pathology and with symptoms potentially related to END prospectively recruited in the above-mentioned divisions prior to surgical intervention, from November 2021 to March 2024. The inclusion criteria for both the retrospective and prospective cohorts were the histological confirmation of either END or non-END benign gynecological disease. The exclusion criteria encompassed a history of malignant tumors, both previous and concurrent, as well as the existence of autoimmune disorders.

All participants signed a written informed-consent form. The research was carried out in compliance with the Declaration of Helsinki and received approval from the Research Review Boards and Ethics Committees of Brescia (study reference number: NP5015) and “Provincia di Venezia e IRCCS San Camillo” (Freedom Trial; study number: 1404).

All blood samples were processed in accordance with the standard operating procedures previously described by Ravaggi *et al.* (2024). The serum aliquots were stored at -80°C and remained frozen until sample processing.

### Isolation of RNA

RNA extraction was conducted by allowing the samples to thaw at ambient temperature. Total RNA was extracted from 400 μl of serum using the miRNeasy Serum/Plasma Advanced Kit (Qiagen, Hilden, Germany) in accordance with the manufacturer’s instructions. RNA was eluted in 22 μl of nuclease-free water and quantified using the Qubit microRNA assay kit (Thermo Fisher Scientific, Waltham, MA, USA) and the Qubit 4 fluorometer (Thermo Fisher Scientific).

### Analysis of target miRNAs with RT-qPCR

For cDNA synthesis, 10 ng of RNA were reverse transcribed in accordance with the manufacturer’s instructions (miRCURY LNA RT Kit, Qiagen). The cDNA was diluted 1:50, and 4 µl were PCR-amplified using a CFX96 TouchTM Real-Time PCR Detection System (Bio-Rad, Hercules, CA, USA), employing iTaq Universal SYBR Green Supermix (Bio-Rad) and specific miRNA PCR primer sets (miRCURY LNA miRNA PCR Assay, Qiagen) (see Supplementary Table S1). Thermal cycling parameters included an initial heat activation of PCR at 95 °C for 10 min, denaturation at 95 °C for 10 s, and a combined annealing/extension at 60 °C for 60 s, repeated for a total of 40 cycles for all primer pairs. All reactions were performed in duplicate. Any sample showing discrepancies between duplicates was retested in triplicate. A melting curve was constructed for each primer pair to verify the specificity of the amplification products. Each plate includes a single inter-run calibration sample to correct for technical variability between multiple runs and to allow comparison of results among plates.

### Statistical analysis

#### Examination of differentially expressed miRNAs

Comparisons between groups (END vs CTR) were conducted using relative quantifications, defined as the ratio of the target miRNA to the arithmetic mean of the most stable miRNAs between groups (reference miRNA) based on RefFinder and TOST, as detailed in the “Materials and methods” section of our prior publication (Ravaggi *et al.*, 2024). When converted to a log_2_ scale, this result equates to -deltaCt. Due to the presence of values below the detection threshold, we adopted a censored regression model approach, because we are unable to measure the exact value of the missing data, but we can get partial information from the threshold (censoring). Models, fitted at the individual miRNA level, were reported as Fold Change (FC), the associated 95% confidence interval (95% CI), and *P*-value.

#### Definition of diagnostic models

Diagnostic models were constructed using two machine learning algorithms: Random Forest (RF) and Logistic Regression (LR), using default tuning parameters. The performance assessment of the various models was derived through internal validation via repeated cross-validation (5 repetitions, 5 folds). Summary estimates were derived by aggregating the estimates from the individual iterations. AUC was adopted as the primary performance metric for a binary or multi-class classification method (Fawcett, 2001). All machine learning models considered perform classification based on probability estimates, using a default setting of 50% to assign the estimated output class. We did not tune the threshold but used the default approach. Based on comparative performance across iterations, RF emerged as the preferred algorithm. A further benefit of using RF is the capacity to readily compute feature importance scores, which facilitate feature selection, as well as the automatic execution of a method for missing data imputation during model fitting.

The employed metrics included AUC (binary or multiclass), sensitivity, specificity, false negative rate (FNR), false positive rate (FPR), and accuracy (ACC). Confidence intervals for cross-validated AUC estimates were computed as in LeDell *et al.* (2015). An iterative removal algorithm, Recursive Feature Elimination (RFE) (Guyon *et al.*, 2002), was employed to assess if a subset of features could enhance performance. RFE evaluates the miRNA set and eliminates the least contributory miRNA to the classification, evaluated using AUC. RFE, as a greedy approach for identifying nested subsets of features, is considered significantly more resilient to data overfitting compared to other approaches. The method underwent cross-validation using 5 folds, repeated five times.

All analyses were conducted using R (https://www.R-project.org/), version 4.5.0.

## Results

### Description of the patient cohort

A total of 136 patients were prospectively enrolled at the two clinical centers; 17 were excluded due to histopathology findings that did not meet the inclusion criteria. Consequently, 82 END patients and 37 CTR patients were added to the 245 participants enrolled retrospectively, resulting in a total of 207 END and 157 CTR patients. The clinicopathologic characteristics of the patients are summarized in Table 1.

**Table 1.** Patient characteristics.

	Controls	Endometriosis
**N. of patients**	157	207
**Age—in years [Mean (SD)]**	38.7 (10.1)	38.5 (8.0)
**BMI [Mean (SD)]**	23.7 (4.7)	22.8 (4.9)

	N. (%)	N. (%)
**Smoke**	33 (21.0)	53 (25.6)
**DIE**	–	84 (40.6)
**OMA**	–	123 (59.4)
**Cystadenoma**	51 (32.5)	–
**Simple serous cyst**	19 (12.1)	–
**Teratoma**	28 (17.8)	–
**Dermoid cyst**	6 (3.8)	–
**Leiomyoma**	31 (19.7)	–
**Other gynecological disorders**	8 (5.1)	–
**No abnormality**	14 (8.9)	–

BMI, body mass index; DIE, deep infiltrating endometriosis; OMA, ovarian endometrioma.

### Study design

**Figure 1.**
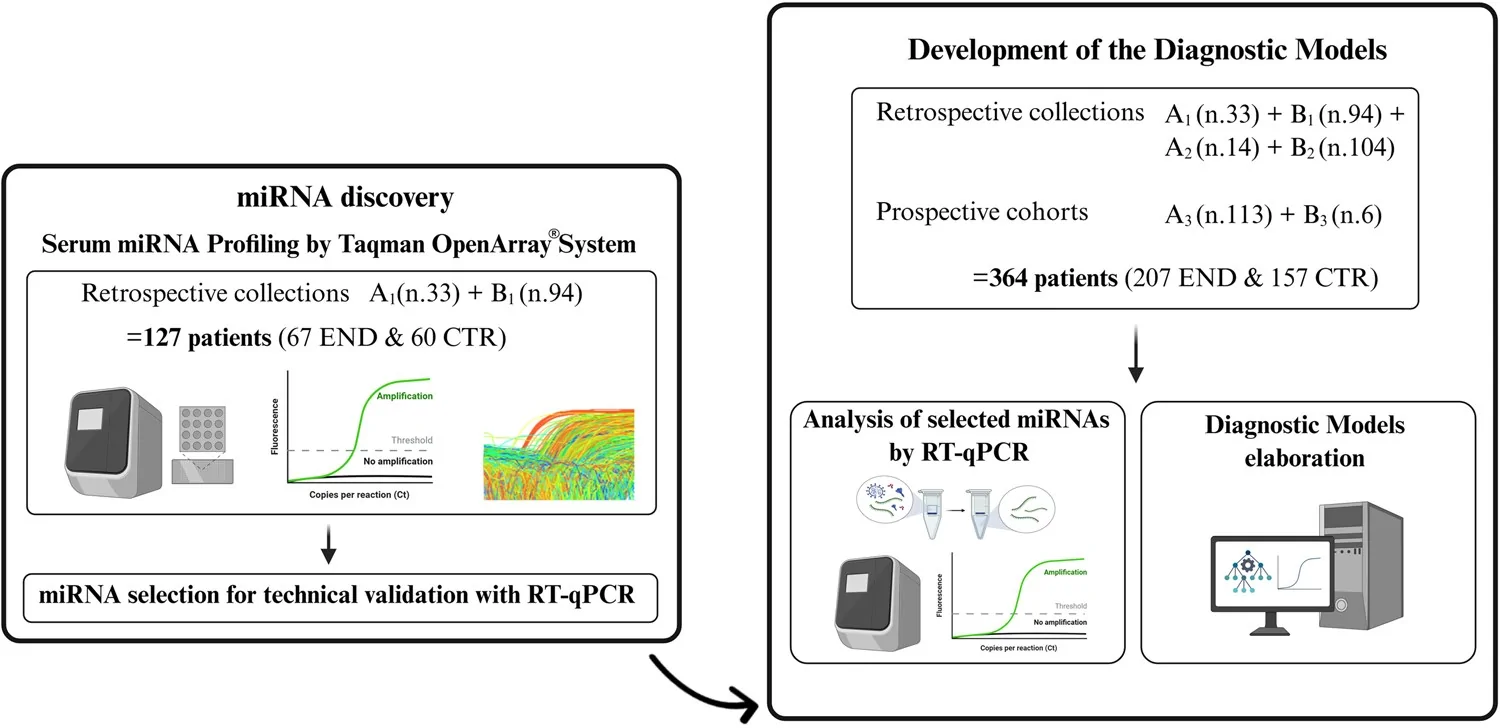
**Schematic representation of the study.** Collection A1, A2, and A3 are from “ASST-Spedali Civili di Brescia”; Collection B1, B2, and B3 are from “Azienda ULSS3 Serenissima”. Created in BioRender. Ferrari, F. (2025) https://BioRender.com/irim6td.

The research was structured in two phases, a first “discovery” phase and a subsequent phase focused on development and internal validation of diagnostic models. In a recent publication by our research group (Ravaggi *et al.*, 2024), we identified miRNAs with different expression levels in END (n = 67) and CTR (n = 60) through OpenArray technology (discovery phase). In the present study, several miRNAs with diagnostic potential were evaluated by RT-qPCR in a larger cohort of 364 patients (207 END and 157 CTR), including those analyzed in the discovery phase. The aim of the present investigation was to create models capable of diagnosing END or END subgroups (DIE and OMA) through cross-validated machine learning algorithms. The study’s design is summarized by the schematic diagram illustrated in Fig. 1.

### Development of a diagnostic model with OpenArray data

A total of 130 miRNAs were identified in at least 75% of serum samples using TaqMan OpenArray Technology in 127 samples (67 END and 60 CTR), as reported in detail in our recent publication (Ravaggi *et al.*, 2024). A diagnostic model was developed from 130 miRNAs using the RF algorithm combined with RFE, focusing on 16 miRNAs (miR-106b-3p, miR-140-3p, miR-181a-5p, miR-181c-5p, miR-22-3p, miR-26a-5p, miR-30b-5p, miR-335-5p, miR-338-3p, miR-340-5p, miR-342-3p, miR-376a-3p, miR-421, miR-485-3p, miR-486-5p, miR-548a-3p) that yielded an accuracy of 69% in differentiating END from CTR. The diagnostic efficacy of this cross-validated model was defined by an AUC of 78%, a FPR of 35%, a FNR of 23%, a specificity of 65%, and a sensitivity of 77%.

### Evaluation of selected miRNAs by RT-qPCR

According to the OpenArray data analysis (Ravaggi *et al.*, 2024), a set of 23 candidate miRNAs was chosen for technical validation via RT-qPCR in the entire cohort of patients, based on the following criteria: (i) the three most invariant miRNAs between END and CTR as potential reference for normalization (miR-15a-5p, miR-26b-5p, and miR-92a-3p); (ii) the 16 miRNAs from the previously mentioned “diagnostic model” (miR-106b-3p, miR-140-3p, miR-181a-5p, miR-181c-5p, miR-22-3p, miR-26a-5p, miR-30b-5p, miR-335-5p, miR-338-3p, miR-340-5p, miR-342-3p, miR-376a-3p, miR-421, miR-485-3p, miR-486-5p, miR-548a-3p); and (iii) four miRNAs that were differentially expressed between END and CTR (miR-92b-3p, miR-29a-3p, miR-652-3p, miR-192-5p,) (Ravaggi *et al.*, 2024).

The three most invariant miRNAs (miR-15a-5p, miR-26b-5p, miR-92a-3p), found from OpenArray results, were confirmed by RT-qPCR and used as reference miRNAs for data normalization. A median Cq value under 30 was considered a reliable detection for the estimation of circulating miRNA (Romani *et al.*, 2021). According to this criterion, six miRNAs (miR-92b-3p, miR-106-3p, miR-181c-5p, miR-421, miR-485-3p, miR-548a-3p) were not considered for further analysis because their Cq values were >30. The findings of the remaining 14 miRNAs are presented in Table 2. Six miRNAs (miR-26a-5p, miR-342-3p, miR-335-5p, miR-181a-5p, miR-30b-5p, miR-340-5p) were found to be significantly underexpressed and one (miR-486-5p) overexpressed in END compared to CTR. The remaining seven miRNAs were significantly differentially expressed only in the comparison between one of the two END subgroups and the CTR; four miRNAs were all down-regulated in DIE (miR-376a-3p, miR-338-3p, miR-29a-3p, miR-652-3p), and two miRNAs were down-regulated (miR-192-5p, miR-140-3p) and one up-regulated (miR-22-3p) in OMA compared to CTR.

**Table 2.** Differential expression of miRNAs in patients with endometriosis compared to controls (CTR), taking into account also all endometriosis (END) lesion subtypes: deep infiltrating endometriosis (DIE) and ovarian endometrioma (OMA).

	END vs CTR			DIE vs CTR			OMA vs CTR		
miRNA	FC	*P*-value	FDR	FC	*P*-value	FDR	FC	*P*-value	FDR
miR-26a-5p	0.76	**<0.01**	<0.01	0.67	**<0.01**	<0.01	0.81	**<0.01**	0.023
miR-342-3p	0.80	**<0.01**	<0.01	0.79	**<0.01**	<0.01	0.81	**<0.01**	<0.01
miR-335-5p	0.80	**<0.01**	<0.01	0.70	**<0.01**	<0.01	0.87	**0.033**	0.066
miR-181a-5p	0.85	**<0.01**	<0.01	0.82	**<0.01**	<0.01	0.87	**<0.01**	0.021
miR-30b-5p	0.88	**<0.01**	<0.01	0.85	**<0.01**	<0.01	0.90	**0.012**	0.038
miR-340-5p	0.79	**<0.01**	<0.01	0.55	**<0.01**	<0.01	0.97	0.695	0.811
miR-486-5p	1.18	**0.012**	0.024	1.45	**<0.01**	<0.01	1.06	0.375	0.583
miR-376a-3p	0.86	0.099	0.139	0.67	**<0.01**	<0.01	0.99	0.943	0.943
miR-338-3p	0.90	0.261	0.281	0.74	**0.010**	0.013	1.02	0.867	0.934
miR-29a-3p	0.89	0.058	0.101	0.79	**<0.01**	<0.01	0.96	0.548	0.715
miR-652-3p	0.94	0.127	0.162	0.81	**<0.01**	<0.01	1.02	0.561	0.715
miR-192-5p	0.91	0.065	0.101	0.99	0.827	0.827	0.86	**0.014**	0.038
miR-140-3p	0.94	0.186	0.217	1.02	0.765	0.824	0.89	**0.026**	0.060
miR-22-3p	1.06	0.319	0.319	0.94	0.395	0.461	1.13	**0.039**	0.069

FC, Fold Change; FDR, false discovery rate. Significant *P*-value <0.05 are in bold.

### Development and internal validation of miRNA-based diagnostic models from RT-qPCR data

To identify the optimal model for diagnosing END, DIE, or OMA in comparison to CTR, both RF and LR methodologies with cross-validation were employed. The two machine learning approaches for each comparison generated 105 models, distinguished by a variable number of miRNAs, spanning from 1 to 14 (Supplementary Tables S2, S3, S4, S5, S6, and S7). Table 3 presents the metrics of the best models derived from the two techniques, revealing that in all three comparisons, the models generated using the cross-validated RF method had superior diagnostic performance compared to those obtained by LR. The RF model based on 11 miRNAs (miR-140-3p, miR-181a-5p, miR-192-5p, miR-22-3p, miR-29a-3p, miR-30b-5p, miR-338-3p, miR-340-5p, miR-342-3p, miR-486-5p, and miR-652-3p) showed an AUC of 70.4%, a sensitivity of 75.6%, and a specificity of 53.5% in differentiating END from CTR.

**Table 3.** Diagnostic performance of the best models built by cross-validated logistic regression (LR) and random forest (RF) algorithms to identify all END patients or END subgroups (DIE, OMA) compared to controls.

Model		miRNAs	AUC %	AUC 95%CI	Sens. %	Spec. %	FNR %	FPR %	ACC %
LR	END vs CTR	miR-140-3p, miR-192-5p, miR-22-3p, miR-26a-5p, miR-342-3p, miR-486-5p, miR-652-3p	67.5	64.9-70.0	75.6	48.7	24.4	51.3	63.8
RF	END vs CTR	miR-140-3p, miR-181a-5p, miR-192-5p, miR-22-3p, miR-29a-3p, miR-30b-5p, miR-338-3p, miR-340-5p, miR-342-3p, miR-486-5p, miR-652-3p	70.4	67.7-73.1	75.6	53.5	24.4	46.5	65.8
LR	DIE vs CTR	miR-192-5p, miR-22-3p, miR-335-5p, miR-340-5p, miR-652-3p	77.9	75.1-80.7	48.4	86.3	51.6	13.7	73.3
RF	DIE vs CTR	miR-192-5p, miR-30b-5p, miR-335-5p, miR-338-3p, miR-486-5p, miR-652-3p	80.4	77.9-82.9	50.9	89.8	49.1	10.2	75.9
LR	OMA vs CTR	miR-181a-5p, miR-342-3p	65.4	62.5-68.3	36.3	79.0	63.7	21.0	59.7
RF	OMA vs CTR	miR-140-3p, miR-26a-5p, miR-30b-5p, miR-338-3p, miR-342-3p, miR-486-5p, miR-652-3p	65.8	62.7-68.9	51.4	70.5	48.6	29.5	62.4

END, all endometriosis; CTR, controls; DIE, deep infiltrating endometriosis; OMA, ovarian endometrioma; 95% CI, 95% confidence interval; Sens., sensitivity; Spec., specificity; FNR, false negative rate; FPR, false positive rate; ACC, accuracy.

The models generated to diagnose the two subgroups of END were characterized by higher specificity compared to the previous model, especially for DIE, with the RF model based on six miRNAs (miR-192-5p, miR-30b-5p, miR-335-5p, miR-338-3p, miR-486-5p, and miR-652-3p) achieving an AUC of 80.4%, a specificity of 89.8%, and a sensitivity of 50.9%. Despite having a higher specificity (70.5%), the RF model based on seven miRNAs (miR-140-3p, miR-26a-5p, miR-30b-5p, miR-338-3p, miR-342-3p, miR-486-5p, miR-652-3p) showed a lower AUC (65.8%) and lower sensitivity (51.4%) for the detection of OMA.

## Discussion

In the present study, we investigated the potential role of serum miRNAs as non-invasive biomarkers for the diagnosis of END. In the first part of the study, we developed a diagnostic model based on 16 miRNAs using OpenArray data published recently by our group (Ravaggi *et al.*, 2024). We then subsequently selected a set of 23 miRNAs for technical validation by RT-qPCR across the entire cohort of enrolled patients. Only a subset of the miRNAs was confirmed by RT-qPCR. These findings may be explained by the substantial increase in the patient cohort size and by methodological differences, particularly the use of pre-amplification in the OpenArray technique but not in RT-qPCR, which may have influenced the observed fold changes. Fourteen out of 23 miRNAs showed significant differences in expression levels when comparing all types of END versus CTR or the two END subtypes (DIE and OMA) versus CTR.

Using qPCR results, we developed several miRNA-based diagnostic models to identify the optimal approach for diagnosing all END cases, as well as DIE or OMA specifically, compared to CTR. Two machine learning methods, RF and LR, with internal cross-validation, were used to create the diagnostic algorithms. The RF model based on 11 miRNAs showed the best AUC (70.4%) in distinguishing all END cases from CTR, with good sensitivity (75.6%) but low specificity (53.5%). Several studies have investigated the potential role of miRNAs as diagnostic biomarkers for END, with conflicting results that are difficult to reproduce since there are many differences in study design, data collection, sample storage, screening techniques, normalization strategies, and statistical approaches. The diagnostic performance of our RF model is consistent with those found in studies involving an adequate number of patients (at least 100). Vanhie *et al.* (2019) identified a set of 42 miRNAs discriminating women with and without END using small RNA sequencing. Three diagnostic models were developed: (i) one to distinguish CTR from all END patients; (ii) another to discriminate between women with minimal-mild END (stage I–II) and CTR; and (iii) a third to identify patients with moderate-severe END (stage III–IV) compared to CTR. After a validation step on an independent cohort of patients, only the second model showed an AUC greater than 50% (AUC = 60%), with acceptable sensitivity (78%) but very low specificity (37%). A study by Nisenblat *et al.* (2019) evaluated the diagnostic potential of the combination of miR-155, miR-574-3p, and miR-139-3p. This model showed an AUC of 70.5%, with sensitivity and specificity values of 83% and 51%, respectively. Pateisky *et al.* (2018), using ROC curve analysis, reported that the combination of four miRNAs (miR-154-5p, miR-196b-5p, miR-378a-3p, miR-33a-5p), differentially expressed in the plasma of END patients compared to CTR, resulted in an AUC of 72%. On the other hand, some studies report miRNA-based algorithms with higher diagnostic performance than those in our study and in the aforementioned studies. In fact, Moustafa *et al.* (2020) identified a signature of six miRNAs (miR-125b-5p, miR-150-5p, miR-342-3p, miR-451a, miR-3613-5p, let-7b) distinguishing END patients from those with benign gynecological disorders with an AUC of 94%. Bendifallah *et al.* (2022b) developed a miRNA-based signature for END diagnosis using small RNA sequencing, obtaining an AUC of 98.4%, a sensitivity of 96.8%, and a specificity of 100%. However, it is important to emphasize that despite the higher performances, there are some critical issues in these studies. These issues include the lack of a validation cohort tested with RT-qPCR, a cost-effective method applicable in a routine diagnostic laboratory (Bendifallah *et al.*, 2022b), and the use of human U6 small nuclear RNA as a reference for RT-qPCR data normalization (Moustafa *et al.*, 2020), despite consolidated evidence of its high variability and instability in studies involving circulating miRNAs (Xiang *et al.*, 2014; Schwarzenbach *et al.*, 2015). Moreover, both studies scored END severity according to the revised American Society of Reproductive Medicine classification, not reporting the percentage of DIE and OMA in their END cohorts. It is important to note that the diagnostic performance of miRNA-based models may be strongly influenced by END subtypes. Indeed, our present findings, along with our previous study (Ravaggi *et al.*, 2024), showed a clear difference in the levels of circulating miRNAs between DIE and OMA compared to CTR, with DIE profiles being more distinct from CTR compared to OMA. One of the strengths of our study is the inclusion of a balanced number of DIE and OMA cases that allowed us to develop diagnostic models potentially identifying not only all END patients but also their subgroups compared to CTR. In particular, the RF model for DIE showed better diagnostic performance than the models for OMA or for all END when compared to CTR. One possible explanation may lie in the biology of DIE lesions, characterized by a higher degree of fibrosis, inflammation, and deeper tissue invasion, which may result in a more distinct miRNA expression profile detectable in peripheral blood compared to CTR. In contrast, OMA are more localized and encapsulated, possibly leading to a weaker systemic miRNA signal. Although the precise molecular mechanisms remain to be fully elucidated, in our study, several miRNAs—miR-340-5p, miR-376a-3p, miR-338-3p, miR-29a-3p, miR-652-3p, and miR-486-5p—showed significant differential expression between DIE and CTR, but not between OMA and CTR, as reported in Table 2. This suggests that DIE lesions are characterized by specific molecular features, different from OMA lesions. All these miRNAs have been previously described in the literature as regulators of pathways relevant to DIE pathogenesis, such as cell proliferation, invasion, epithelial–mesenchymal transition (EMT), and angiogenesis. For instance, downregulation of miR-340-5p promotes proliferation, invasion, and EMT by activating MAPK/ERK signaling via MAP3K2 in END (Wan *et al.*, 2022). MiR-486-5p overexpression promotes cell proliferation, migration, and invasion by suppressing PTEN expression and activating the PI3K/Akt signaling pathway in cervical cancer (Li *et al.*, 2018) and targeting MARK1 in endometrial cancer (Zheng *et al.*, 2020). MiR-376a-3p is known as a tumor suppressor in several cancer types, and its downregulation leads to the overexpression of genes involved in cell-cell communication, EMT, angiogenesis, and mTOR-mediated signaling (Zhang *et al.*, 2018). MiR-338-3p acts predominantly as a tumor suppressor in various cancers by modulating WNT, MAPK, and PI3K/AKT pathways, and its downregulation is linked to enhanced cell proliferation, migration, and angiogenesis (Moghbeli, 2021). MiR-29a-3p downregulation increases migration and proliferation in HeLa cells through de-repression of SNIP1 and downstream effectors such as c-Myc and cyclinD1 (Chen *et al.*, 2020) and has also been implicated as a tumor suppressor in gastric cancer (Zhao *et al.*, 2015). Finally, reduced expression of miR-652-3p was found to promote cancer cell proliferation, invasion, and EMT in pancreatic cancer by targeting ZEB1 (Deng *et al.*, 2015). Although these findings are derived primarily from cancer models, they suggest that similar mechanisms could potentially be active in DIE lesions. However, to date, no studies have demonstrated these pathways in DIE, and they would need to be confirmed at the experimental level.

Our RF models, generated to diagnose DIE and OMA, were characterized by high specificity but low sensitivity. This discrepancy highlights the trade-off often observed between sensitivity and specificity in diagnostic models. While higher specificity can reduce false positive cases, it may also reduce sensitivity, leading to missed diagnoses, particularly for OMA cases. However, the diagnosis of OMA by means of transvaginal ultrasound can achieve a sensitivity of 93% if performed by an experienced sonographer. On the contrary, the ultrasound diagnosis of DIE is more complex, and the sensitivity varies depending on the location of the lesion, with a range between 52% and 93% (Young *et al.*, 2024).

Regarding the DIE diagnostic model, the high specificity (90%) represents a valuable advantage in a clinical context where the misdiagnosis may lead to unnecessary invasive treatments, potentially affecting the patients’ physical and psychological well-being. Moreover, the model showed an AUC of 80% and an FPR of 10%, indicating that it can correctly identify DIE cases with a low error probability. Although the FNR is relatively high (50%), indicating that some DIE cases might remain undetected, these results still suggest that our DIE diagnostic model could serve as a valuable decision-support instrument for clinicians, potentially representing an initial screening tool to guide patients toward further diagnostic evaluation during the early stages of clinical suspicion. Furthermore, this higher specificity in DIE diagnosis has significant clinico-surgical value; in fact, often during the surgical intervention of patients with OMA, the presence of DIE is unexpectedly found, requiring a different and more complex surgical approach. A laboratory test, combined with imaging, could accurately assess the patient’s condition and refer them to a specialized END center. Among the miRNAs included in our three RF models, six (miR-342-3p, miR-181a-5p, miR-30b-5p, miR-340-5p, miR-26a-5p, miR-335-5p) were significantly downregulated, and one (miR-486-5p) was upregulated in all END patients compared to CTR. Regarding miR-342-3p, the literature reports associations with END, although findings are conflicting (Cosar *et al.*, 2016; Wang *et al.*, 2016; Moustafa *et al.*, 2020). Consistent with our results, Pateisky *et al.* (2018) reported that miR-181a-5p was downregulated in plasma samples from women with END compared to CTR. MiR-30b-5p has also been previously reported in several studies (Wang *et al.*, 2016; Chico-Sordo *et al.*, 2024) as differentially expressed in the serum of END patients. Bahramy *et al.* (2021) and Papari *et al.* (2020) described the downregulation of miR-340-5p in plasma samples from women with END compared to CTR, in agreement with our findings. Both miR-26a-5p and miR-335-5p were found to be differentially expressed in a study by Wang *et al.* (2016). Moreover, miR-26a-5p was included in a miRNA panel, which was able to differentiate END patients from healthy women (Vanhie *et al.*, 2019). Regarding the upregulated miR-486-5p, Marí-Alexandre *et al.* (2018) previously reported its upregulation in the peritoneal fluid of women with END.

Among the main strengths of our investigation, there is the multicentric design of the study and the large and well-balanced patient cohort, not only between END and CTR but also across the different END subtypes. According to the literature (Steyerberg and Harrell, 2016), proper validation should include internal, internal–external, and external validation steps to robustly assess model performance and generalizability. In our study, we decided to use the entire retrospective cohort, including discovery-phase patients, together with the prospectively enrolled cohort for both model development and internal cross-validation. This choice was driven by sample size limitations within individual sub-cohorts. This strategy enabled us to maximize statistical power and exploit the full heterogeneity of our sample. In addition, the inclusion of data derived from the prospective cohort offers a partial internal–external validation framework, enhancing the robustness of our results within the limits of available data. Other strengths of our work include the standardization of the pre-analytical procedures, the surgical and histological confirmation of diagnosis for all enrolled patients, and finally, the quantification of miRNA extracted from serum, which improved the accuracy of our results. Another key positive aspect of our study lies in the normalization method we adopted, in that the most stable miRNAs among the groups were selected as reference miRNAs based on OpenArray data. These results were also confirmed by RT-qPCR, which provided a double-level validation that enhanced the robustness and reliability of our normalization approach.

### Limitations and challenges

Despite several strengths, the main limitation of our study was the lack of external validation using an independent cohort. Although we applied a repeated cross-validation to internally validate the model and reduce overfitting, the use of the same dataset for both training and validation might still have led to optimistic performance estimates. Moreover, as reported in our recent publication (Ravaggi *et al.*, 2024), due to the partial retrospective nature of the study, some information, such as END stage and the menstrual cycle phase at the time of serum sample collection, was unavailable for many patients. Although several studies have reported that circulating miRNA levels in the blood of END patients are not influenced by the hormonal fluctuations associated with different phases of the menstrual cycle (Wang *et al.*, 2013; Cosar *et al.*, 2016; Nisenblat *et al.*, 2019; Vanhie *et al.*, 2019; Moustafa *et al.*, 2020; Chico-Sordo *et al.*, 2024) or CTR (Rekker *et al.*, 2013), we cannot entirely exclude the possibility that some miRNAs may still be sensitive to such variations. This could potentially affect the diagnostic performance of the model if not properly accounted for.

### Future perspective

In future studies, we plan to investigate whether incorporating information on the hormonal phase of patients at the time of enrolment may influence the diagnostic accuracy of the model. To this aim, we intend to include in the analysis the quantification of FSH, LH, oestradiol, and progesterone as indicators of the menstrual phase. Moreover, we plan to validate these diagnostic models on independent prospective cohorts of patients to confirm the generalizability and clinical utility of our findings.

## Supplementary Material

deaf221_Supplementary_Table_S1

deaf221_Supplementary_Table_S2

deaf221_Supplementary_Table_S3

deaf221_Supplementary_Table_S4

deaf221_Supplementary_Table_S5

deaf221_Supplementary_Table_S6

deaf221_Supplementary_Table_S7

## Data Availability

Raw data of miRNA expression obtained by OpenArray technology have been deposited in NCBI’s Gene Expression Omnibus and are accessible through GEO Series accession numbers GSE279435. The other data could be shared upon reasonable request to the corresponding author.
