# Supplementary material for: Serum miRNA-based diagnostic models for endometriosis: from discovery to validation
Source: Hum Reprod. 2025 Nov 21;41(2):195–203. doi: 10.1093/humrep/deaf221 (PMC12864148; doi:10.1093/humrep/deaf221)
Supplement: deaf221_Supplementary_Table_S1 [file deaf221_supplementary_table_s1.pdf]

OXFORD
human reproduction

Human Reproduction, 2025, 1–1
https://doi.org/10.1093/humrep/deaf221
Supplementary Data

Supplementary Table S1. Characteristics of selected miRNAs for validation by RT-qPCR.

miRNA	Sequence	Access number
hsa-miR-103a-3p	AGCAGCAUUGUACAGGGCUAUGA	MIMAT0000101
hsa-miR-106b-3p	CCGCACUGUGGGUACUUGCUGC	MIMAT0004672
hsa-miR-140-3p	UACCACAGGGUAGAACCACGG	MIMAT0004597
hsa-miR-143-3p	UGAGAUGAAGCACUGUAGCUC	MIMAT0000435
hsa-miR-15a-5p	UAGCAGCACAUAAUGGUUUGUG	MIMAT0000068
hsa-miR-17-3p	ACUGCAGUGAAGGCACUUGUAG	MIMAT0000071
hsa-miR-181a-5p	AACAUUCAACGCUGUCGGUGAGU	MIMAT0000256
hsa-miR-181c-5p	AACAUUCAACCUGUCGGUGAGU	MIMAT0000258
hsa-miR-192-5p	CUGACCUAUGAAUUGACAGCC	MIMAT0000222
hsa-miR-22-3p	AAGCUGCCAGUUGAAGAACUGU	MIMAT0000077
hsa-miR-26a-5p	UUCAAGUAAUCCAGGAUAGGCU	MIMAT0000082
hsa-miR-26b-5p	UUCAAGUAAUUCAGGAUAGGU	MIMAT0000083
hsa-miR-29a-3p	UAGCACCAUCUGAAAUCGGUUA	MIMAT0000086
hsa-miR-30b-5p	UGUAAACAUCCUACACUCAGCU	MIMAT0000420
hsa-miR-335-5p	UCAAGAGCAAUAACGAAAAAUGU	MIMAT0000765
hsa-miR-338-3p	UCCAGCAUCAGUGAUUUUGUUG	MIMAT0000763
hsa-miR-340-5p	UUAUAAAGCAAUGAGACUGAUU	MIMAT0004692
hsa-miR-342-3p	UCUCACACAGAAAUCGCACCCGU	MIMAT0000753
hsa-miR-376a-3p	AUCAUAGAGGAAAAUCCACGU	MIMAT0000729
hsa-miR-421	AUCAACAGACAUUAAUUGGGCGC	MIMAT0003339
hsa-miR-485-3p	GUCAUACACGGCUCUCCUCUCU	MIMAT0002176
hsa-miR-486-5p	UCCUGUACUGAGCUGCCCCGAG	MIMAT0002177
hsa-miR-548a-3p	CAAAACUGGCAAUUACUUUUGC	MIMAT0003251
hsa-miR-652-3p	AAUGGCGCCACUAGGGUUGUG	MIMAT0003322
hsa-miR-92a-3p	UAUUGCACUUGUCCCGGCCUGU	MIMAT0000092
hsa-miR-92b-3p	UAUUGCACUCGUCCCGGCCUCC	MIMAT0003218
