# Supplementary material for: Serum miRNA-based diagnostic models for endometriosis: from discovery to validation
Source: Hum Reprod. 2025 Nov 21;41(2):195–203. doi: 10.1093/humrep/deaf221 (PMC12864148; doi:10.1093/humrep/deaf221)
Supplement: deaf221_Supplementary_Table_S2 [file deaf221_supplementary_table_s2.pdf]

OXFORD
human reproduction
Human Reproduction, 2025, 1–2
https://doi.org/10.1093/humrep/deaf221
Supplementary Data

Supplementary Table S2. Diagnostic models built by the logistic regression (LR) algorithm to differentiate endometriosis patients (END) from controls (CTR).

LR models: END vs CTR	AUC
miR-140-3p	54.11
miR-181a-5p	62.05
miR-192-5p	55.81
miR-22-3p	52.96
miR-26a-5p	63.32
miR-29a-3p	56.64
miR-30b-5p	59.99
miR-335-5p	60.89
miR-338-3p	47.53
miR-340-5p	59.66
miR-342-3p	62.69
miR-376a-3p	55.52
miR-486-5p	57.55
miR-652-3p	53.77
miR-140-3p, miR-26a-5p	62.92
miR-181a-5p, miR-26a-5p	64.26
miR-192-5p, miR-26a-5p	63.37
miR-22-3p, miR-26a-5p	62.52
miR-26a-5p, miR-29a-3p	62.93
miR-26a-5p, miR-30b-5p	62.75
miR-26a-5p, miR-335-5p	62.54
miR-26a-5p, miR-338-3p	62.45
miR-26a-5p, miR-340-5p	63.09
miR-26a-5p, miR-342-3p	64.68
miR-26a-5p, miR-376a-3p	62.70
miR-26a-5p, miR-486-5p	62.64
miR-26a-5p, miR-652-3p	63.43
miR-140-3p, miR-26a-5p, miR-342-3p	64.26
miR-181a-5p, miR-26a-5p, miR-342-3p	65.67
miR-192-5p, miR-26a-5p, miR-342-3p	64.37
miR-22-3p, miR-26a-5p, miR-342-3p	63.94
miR-26a-5p, miR-29a-3p, miR-342-3p	64.47
miR-26a-5p, miR-30b-5p, miR-342-3p	64.08
miR-26a-5p, miR-335-5p, miR-342-3p	63.88
miR-26a-5p, miR-338-3p, miR-342-3p	63.92
miR-26a-5p, miR-340-5p, miR-342-3p	64.89
miR-26a-5p, miR-342-3p, miR-376a-3p	64.11
miR-26a-5p, miR-342-3p, miR-486-5p	64.96
miR-26a-5p, miR-342-3p, miR-652-3p	66.31
miR-140-3p, miR-26a-5p, miR-342-3p, miR-652-3p	66.01
miR-181a-5p, miR-26a-5p, miR-342-3p, miR-652-3p	65.99
miR-192-5p, miR-26a-5p, miR-342-3p, miR-652-3p	66.50
miR-22-3p, miR-26a-5p, miR-342-3p, miR-652-3p	66.83
miR-26a-5p, miR-29a-3p, miR-342-3p, miR-652-3p	65.59
miR-26a-5p, miR-30b-5p, miR-342-3p, miR-652-3p	65.54
miR-26a-5p, miR-335-5p, miR-342-3p, miR-652-3p	65.36
miR-26a-5p, miR-338-3p, miR-342-3p, miR-652-3p	65.85
miR-26a-5p, miR-340-5p, miR-342-3p, miR-652-3p	65.49
miR-26a-5p, miR-342-3p, miR-376a-3p, miR-652-3p	66.10
miR-26a-5p, miR-342-3p, miR-486-5p, miR-652-3p	65.56
miR-140-3p, miR-22-3p, miR-26a-5p, miR-342-3p, miR-652-3p	66.66
miR-181a-5p, miR-22-3p, miR-26a-5p, miR-342-3p, miR-652-3p	66.43
miR-192-5p, miR-22-3p, miR-26a-5p, miR-342-3p, miR-652-3p	67.05
miR-22-3p, miR-26a-5p, miR-29a-3p, miR-342-3p, miR-652-3p	66.70
miR-22-3p, miR-26a-5p, miR-30b-5p, miR-342-3p, miR-652-3p	66.24
miR-22-3p, miR-26a-5p, miR-335-5p, miR-342-3p, miR-652-3p	66.27
miR-22-3p, miR-26a-5p, miR-338-3p, miR-342-3p, miR-652-3p	66.18
miR-22-3p, miR-26a-5p, miR-340-5p, miR-342-3p, miR-652-3p	66.36
miR-22-3p, miR-26a-5p, miR-342-3p, miR-376a-3p, miR-652-3p	66.63
miR-22-3p, miR-26a-5p, miR-342-3p, miR-486-5p, miR-652-3p	66.43
miR-140-3p, miR-192-5p, miR-22-3p, miR-26a-5p, miR-342-3p, miR-652-3p	67.06
miR-181a-5p, miR-192-5p, miR-22-3p, miR-26a-5p, miR-342-3p, miR-652-3p	66.54

(continued)

Supplementary Table S2. (continued)

LR models: END vs CTR	AUC
miR-192-5p, miR-22-3p, miR-26a-5p, miR-29a-3p, miR-342-3p, miR-652-3p	67.03
miR-192-5p, miR-22-3p, miR-26a-5p, miR-30b-5p, miR-342-3p, miR-652-3p	66.74
miR-192-5p, miR-22-3p, miR-26a-5p, miR-335-5p, miR-342-3p, miR-652-3p	66.35
miR-192-5p, miR-22-3p, miR-26a-5p, miR-338-3p, miR-342-3p, miR-652-3p	66.48
miR-192-5p, miR-22-3p, miR-26a-5p, miR-340-5p, miR-342-3p, miR-652-3p	66.83
miR-192-5p, miR-22-3p, miR-26a-5p, miR-342-3p, miR-376a-3p, miR-652-3p	66.94
miR-192-5p, miR-22-3p, miR-26a-5p, miR-342-3p, miR-486-5p, miR-652-3p	67.21
**miR-140-3p, miR-192-5p, miR-22-3p, miR-26a-5p, miR-342-3p, miR-486-5p, miR-652-3p**	**67.48**
miR-181a-5p, miR-192-5p, miR-22-3p, miR-26a-5p, miR-342-3p, miR-486-5p, miR-652-3p	67.34
miR-192-5p, miR-22-3p, miR-26a-5p, miR-29a-3p, miR-342-3p, miR-486-5p, miR-652-3p	66.78
miR-192-5p, miR-22-3p, miR-26a-5p, miR-30b-5p, miR-342-3p, miR-486-5p, miR-652-3p	66.79
miR-192-5p, miR-22-3p, miR-26a-5p, miR-335-5p, miR-342-3p, miR-486-5p, miR-652-3p	66.81
miR-192-5p, miR-22-3p, miR-26a-5p, miR-338-3p, miR-342-3p, miR-486-5p, miR-652-3p	66.76
miR-192-5p, miR-22-3p, miR-26a-5p, miR-340-5p, miR-342-3p, miR-486-5p, miR-652-3p	66.39
miR-192-5p, miR-22-3p, miR-26a-5p, miR-342-3p, miR-376a-3p, miR-486-5p, miR-652-3p	66.93
miR-140-3p, miR-181a-5p, miR-192-5p, miR-22-3p, miR-26a-5p, miR-342-3p, miR-486-5p, miR-652-3p	67.40
miR-140-3p, miR-192-5p, miR-22-3p, miR-26a-5p, miR-29a-3p, miR-342-3p, miR-486-5p, miR-652-3p	67.01
miR-140-3p, miR-192-5p, miR-22-3p, miR-26a-5p, miR-30b-5p, miR-342-3p, miR-486-5p, miR-652-3p	66.93
miR-140-3p, miR-192-5p, miR-22-3p, miR-26a-5p, miR-335-5p, miR-342-3p, miR-486-5p, miR-652-3p	67.11
miR-140-3p, miR-192-5p, miR-22-3p, miR-26a-5p, miR-338-3p, miR-342-3p, miR-486-5p, miR-652-3p	67.11
miR-140-3p, miR-192-5p, miR-22-3p, miR-26a-5p, miR-340-5p, miR-342-3p, miR-486-5p, miR-652-3p	66.86
miR-140-3p, miR-192-5p, miR-22-3p, miR-26a-5p, miR-342-3p, miR-376a-3p, miR-486-5p, miR-652-3p	67.21
miR-140-3p, miR-181a-5p, miR-192-5p, miR-22-3p, miR-26a-5p, miR-29a-3p, miR-342-3p, miR-486-5p, miR-652-3p	66.92
miR-140-3p, miR-181a-5p, miR-192-5p, miR-22-3p, miR-26a-5p, miR-30b-5p, miR-342-3p, miR-486-5p, miR-652-3p	66.75
miR-140-3p, miR-181a-5p, miR-192-5p, miR-22-3p, miR-26a-5p, miR-335-5p, miR-342-3p, miR-486-5p, miR-652-3p	66.93
miR-140-3p, miR-181a-5p, miR-192-5p, miR-22-3p, miR-26a-5p, miR-338-3p, miR-342-3p, miR-486-5p, miR-652-3p	67.25
miR-140-3p, miR-181a-5p, miR-192-5p, miR-22-3p, miR-26a-5p, miR-340-5p, miR-342-3p, miR-486-5p, miR-652-3p	67.01
miR-140-3p, miR-181a-5p, miR-192-5p, miR-22-3p, miR-26a-5p, miR-342-3p, miR-376a-3p, miR-486-5p, miR-652-3p	67.20
miR-140-3p, miR-181a-5p, miR-192-5p, miR-22-3p, miR-26a-5p, miR-29a-3p, miR-338-3p, miR-342-3p, miR-486-5p, miR-652-3p	66.92
miR-140-3p, miR-181a-5p, miR-192-5p, miR-22-3p, miR-26a-5p, miR-30b-5p, miR-338-3p, miR-342-3p, miR-486-5p, miR-652-3p	67.38
miR-140-3p, miR-181a-5p, miR-192-5p, miR-22-3p, miR-26a-5p, miR-335-5p, miR-338-3p, miR-342-3p, miR-486-5p, miR-652-3p	66.81
miR-140-3p, miR-181a-5p, miR-192-5p, miR-22-3p, miR-26a-5p, miR-338-3p, miR-340-5p, miR-342-3p, miR-486-5p, miR-652-3p	66.49
miR-140-3p, miR-181a-5p, miR-192-5p, miR-22-3p, miR-26a-5p, miR-338-3p, miR-342-3p, miR-376a-3p, miR-486-5p, miR-652-3p	67.01
miR-140-3p, miR-181a-5p, miR-192-5p, miR-22-3p, miR-26a-5p, miR-29a-3p, miR-30b-5p, miR-338-3p, miR-342-3p, miR-486-5p, miR-652-3p	67.04
miR-140-3p, miR-181a-5p, miR-192-5p, miR-22-3p, miR-26a-5p, miR-30b-5p, miR-335-5p, miR-338-3p, miR-342-3p, miR-486-5p, miR-652-3p	66.70
miR-140-3p, miR-181a-5p, miR-192-5p, miR-22-3p, miR-26a-5p, miR-30b-5p, miR-338-3p, miR-340-5p, miR-342-3p, miR-486-5p, miR-652-3p	66.61
miR-140-3p, miR-181a-5p, miR-192-5p, miR-22-3p, miR-26a-5p, miR-30b-5p, miR-338-3p, miR-342-3p, miR-376a-3p, miR-486-5p, miR-652-3p	67.03
miR-140-3p, miR-181a-5p, miR-192-5p, miR-22-3p, miR-26a-5p, miR-29a-3p, miR-30b-5p, miR-335-5p, miR-338-3p, miR-342-3p, miR-486-5p, miR-652-3p	66.39
miR-140-3p, miR-181a-5p, miR-192-5p, miR-22-3p, miR-26a-5p, miR-29a-3p, miR-30b-5p, miR-338-3p, miR-340-5p, miR-342-3p, miR-486-5p, miR-652-3p	66.23
miR-140-3p, miR-181a-5p, miR-192-5p, miR-22-3p, miR-26a-5p, miR-29a-3p, miR-30b-5p, miR-338-3p, miR-342-3p, miR-376a-3p, miR-486-5p, miR-652-3p	66.75
miR-140-3p, miR-181a-5p, miR-192-5p, miR-22-3p, miR-26a-5p, miR-29a-3p, miR-30b-5p, miR-335-5p, miR-338-3p, miR-342-3p, miR-376a-3p, miR-486-5p, miR-652-3p	66.23
miR-140-3p, miR-181a-5p, miR-192-5p, miR-22-3p, miR-26a-5p, miR-29a-3p, miR-30b-5p, miR-338-3p, miR-340-5p, miR-342-3p, miR-376a-3p, miR-486-5p, miR-652-3p	66.12
miR-140-3p, miR-181a-5p, miR-192-5p, miR-22-3p, miR-26a-5p, miR-29a-3p, miR-30b-5p, miR-335-5p, miR-338-3p, miR-340-5p, miR-342-3p, miR-376a-3p, miR-486-5p, miR-652-3p	65.60

The performance assessment of the various models was derived from internal validation, utilizing repeated cross-validation (5 repetitions, 5 folds).
