# Supplementary material for: Serum miRNA-based diagnostic models for endometriosis: from discovery to validation
Source: Hum Reprod. 2025 Nov 21;41(2):195–203. doi: 10.1093/humrep/deaf221 (PMC12864148; doi:10.1093/humrep/deaf221)
Supplement: deaf221_Supplementary_Table_S3 [file deaf221_supplementary_table_s3.pdf]

OXFORD
human reproduction
Human Reproduction, 2025, 1–2
https://doi.org/10.1093/humrep/deaf221
Supplementary Data

Supplementary Table S3. Diagnostic models built by the Random Forest (RF) algorithm to differentiate endometriosis patients (END) from controls (CTR).

RF models: END vs CTR	AUC
miR-140-3p	52.52
miR-181a-5p	54.27
miR-192-5p	52.55
miR-22-3p	47.46
miR-26a-5p	52.89
miR-29a-3p	59.78
miR-30b-5p	54.80
miR-335-5p	44.42
miR-338-3p	47.06
miR-340-5p	50.36
miR-342-3p	56.01
miR-376a-3p	45.38
miR-486-5p	56.38
miR-652-3p	50.88
miR-140-3p, miR-29a-3p	56.42
miR-181a-5p, miR-29a-3p	57.46
miR-192-5p, miR-29a-3p	57.13
miR-22-3p, miR-29a-3p	55.16
miR-26a-5p, miR-29a-3p	58.32
miR-29a-3p, miR-30b-5p	58.27
miR-29a-3p, miR-335-5p	54.30
miR-29a-3p, miR-338-3p	60.24
miR-29a-3p, miR-340-5p	53.87
miR-29a-3p, miR-342-3p	62.93
miR-29a-3p, miR-376a-3p	57.12
miR-29a-3p, miR-486-5p	55.22
miR-29a-3p, miR-652-3p	54.45
miR-140-3p, miR-29a-3p, miR-342-3p	62.97
miR-181a-5p, miR-29a-3p, miR-342-3p	64.48
miR-192-5p, miR-29a-3p, miR-342-3p	59.30
miR-22-3p, miR-29a-3p, miR-342-3p	61.68
miR-26a-5p, miR-29a-3p, miR-342-3p	62.34
miR-29a-3p, miR-30b-5p, miR-342-3p	63.20
miR-29a-3p, miR-335-5p, miR-342-3p	60.24
miR-29a-3p, miR-338-3p, miR-342-3p	60.86
miR-29a-3p, miR-340-5p, miR-342-3p	59.57
miR-29a-3p, miR-342-3p, miR-376a-3p	60.98
miR-29a-3p, miR-342-3p, miR-486-5p	64.17
miR-29a-3p, miR-342-3p, miR-652-3p	63.08
miR-140-3p, miR-181a-5p, miR-29a-3p, miR-342-3p	65.03
miR-181a-5p, miR-192-5p, miR-29a-3p, miR-342-3p	63.85
miR-181a-5p, miR-22-3p, miR-29a-3p, miR-342-3p	64.22
miR-181a-5p, miR-26a-5p, miR-29a-3p, miR-342-3p	63.25
miR-181a-5p, miR-29a-3p, miR-30b-5p, miR-342-3p	64.29
miR-181a-5p, miR-29a-3p, miR-335-5p, miR-342-3p	61.75
miR-181a-5p, miR-29a-3p, miR-338-3p, miR-342-3p	62.39
miR-181a-5p, miR-29a-3p, miR-340-5p, miR-342-3p	61.97
miR-181a-5p, miR-29a-3p, miR-342-3p, miR-376a-3p	64.97
miR-181a-5p, miR-29a-3p, miR-342-3p, miR-486-5p	65.91
miR-181a-5p, miR-29a-3p, miR-342-3p, miR-652-3p	64.77
miR-140-3p, miR-181a-5p, miR-29a-3p, miR-342-3p, miR-486-5p	66.51
miR-181a-5p, miR-192-5p, miR-29a-3p, miR-342-3p, miR-486-5p	65.72
miR-181a-5p, miR-22-3p, miR-29a-3p, miR-342-3p, miR-486-5p	66.05
miR-181a-5p, miR-26a-5p, miR-29a-3p, miR-342-3p, miR-486-5p	66.02
miR-181a-5p, miR-29a-3p, miR-30b-5p, miR-342-3p, miR-486-5p	64.67
miR-181a-5p, miR-29a-3p, miR-335-5p, miR-342-3p, miR-486-5p	63.94
miR-181a-5p, miR-29a-3p, miR-338-3p, miR-342-3p, miR-486-5p	64.53
miR-181a-5p, miR-29a-3p, miR-340-5p, miR-342-3p, miR-486-5p	65.37
miR-181a-5p, miR-29a-3p, miR-342-3p, miR-376a-3p, miR-486-5p	66.74
miR-181a-5p, miR-29a-3p, miR-342-3p, miR-486-5p, miR-652-3p	66.79
miR-140-3p, miR-181a-5p, miR-29a-3p, miR-342-3p, miR-486-5p, miR-652-3p	68.58
miR-181a-5p, miR-192-5p, miR-29a-3p, miR-342-3p, miR-486-5p, miR-652-3p	66.43

(continued)

Supplementary Table S3. (continued)

RF models: END vs CTR	AUC
miR-181a-5p, miR-22-3p, miR-29a-3p, miR-342-3p, miR-486-5p, miR-652-3p	67.92
miR-181a-5p, miR-26a-5p, miR-29a-3p, miR-342-3p, miR-486-5p, miR-652-3p	67.69
miR-181a-5p, miR-29a-3p, miR-30b-5p, miR-342-3p, miR-486-5p, miR-652-3p	66.48
miR-181a-5p, miR-29a-3p, miR-335-5p, miR-342-3p, miR-486-5p, miR-652-3p	66.14
miR-181a-5p, miR-29a-3p, miR-338-3p, miR-342-3p, miR-486-5p, miR-652-3p	66.28
miR-181a-5p, miR-29a-3p, miR-340-5p, miR-342-3p, miR-486-5p, miR-652-3p	66.87
miR-181a-5p, miR-29a-3p, miR-342-3p, miR-376a-3p, miR-486-5p, miR-652-3p	67.56
miR-140-3p, miR-181a-5p, miR-192-5p, miR-29a-3p, miR-342-3p, miR-486-5p, miR-652-3p	67.63
miR-140-3p, miR-181a-5p, miR-22-3p, miR-29a-3p, miR-342-3p, miR-486-5p, miR-652-3p	68.44
miR-140-3p, miR-181a-5p, miR-26a-5p, miR-29a-3p, miR-342-3p, miR-486-5p, miR-652-3p	67.72
miR-140-3p, miR-181a-5p, miR-29a-3p, miR-30b-5p, miR-342-3p, miR-486-5p, miR-652-3p	67.71
miR-140-3p, miR-181a-5p, miR-29a-3p, miR-335-5p, miR-342-3p, miR-486-5p, miR-652-3p	65.93
miR-140-3p, miR-181a-5p, miR-29a-3p, miR-338-3p, miR-342-3p, miR-486-5p, miR-652-3p	67.27
miR-140-3p, miR-181a-5p, miR-29a-3p, miR-340-5p, miR-342-3p, miR-486-5p, miR-652-3p	68.21
miR-140-3p, miR-181a-5p, miR-29a-3p, miR-342-3p, miR-376a-3p, miR-486-5p, miR-652-3p	67.55
miR-140-3p, miR-181a-5p, miR-192-5p, miR-22-3p, miR-29a-3p, miR-342-3p, miR-486-5p, miR-652-3p	68.51
miR-140-3p, miR-181a-5p, miR-22-3p, miR-26a-5p, miR-29a-3p, miR-342-3p, miR-486-5p, miR-652-3p	68.39
miR-140-3p, miR-181a-5p, miR-22-3p, miR-29a-3p, miR-30b-5p, miR-342-3p, miR-486-5p, miR-652-3p	68.31
miR-140-3p, miR-181a-5p, miR-22-3p, miR-29a-3p, miR-335-5p, miR-342-3p, miR-486-5p, miR-652-3p	68.63
miR-140-3p, miR-181a-5p, miR-22-3p, miR-29a-3p, miR-338-3p, miR-342-3p, miR-486-5p, miR-652-3p	68.44
miR-140-3p, miR-181a-5p, miR-22-3p, miR-29a-3p, miR-340-5p, miR-342-3p, miR-486-5p, miR-652-3p	70.25
miR-140-3p, miR-181a-5p, miR-22-3p, miR-29a-3p, miR-342-3p, miR-376a-3p, miR-486-5p, miR-652-3p	67.84
miR-140-3p, miR-181a-5p, miR-192-5p, miR-22-3p, miR-29a-3p, miR-340-5p, miR-342-3p, miR-486-5p, miR-652-3p	69.71
miR-140-3p, miR-181a-5p, miR-22-3p, miR-26a-5p, miR-29a-3p, miR-340-5p, miR-342-3p, miR-486-5p, miR-652-3p	69.64
miR-140-3p, miR-181a-5p, miR-22-3p, miR-29a-3p, miR-30b-5p, miR-340-5p, miR-342-3p, miR-486-5p, miR-652-3p	69.41
miR-140-3p, miR-181a-5p, miR-22-3p, miR-29a-3p, miR-335-5p, miR-340-5p, miR-342-3p, miR-486-5p, miR-652-3p	68.81
miR-140-3p, miR-181a-5p, miR-22-3p, miR-29a-3p, miR-338-3p, miR-340-5p, miR-342-3p, miR-486-5p, miR-652-3p	69.93
miR-140-3p, miR-181a-5p, miR-22-3p, miR-29a-3p, miR-340-5p, miR-342-3p, miR-376a-3p, miR-486-5p, miR-652-3p	69.91
miR-140-3p, miR-181a-5p, miR-192-5p, miR-22-3p, miR-29a-3p, miR-338-3p, miR-340-5p, miR-342-3p, miR-486-5p, miR-652-3p	69.46
miR-140-3p, miR-181a-5p, miR-22-3p, miR-26a-5p, miR-29a-3p, miR-338-3p, miR-340-5p, miR-342-3p, miR-486-5p, miR-652-3p	68.93
miR-140-3p, miR-181a-5p, miR-22-3p, miR-29a-3p, miR-30b-5p, miR-338-3p, miR-340-5p, miR-342-3p, miR-486-5p, miR-652-3p	70.27
miR-140-3p, miR-181a-5p, miR-22-3p, miR-29a-3p, miR-335-5p, miR-338-3p, miR-340-5p, miR-342-3p, miR-486-5p, miR-652-3p	67.86
miR-140-3p, miR-181a-5p, miR-22-3p, miR-29a-3p, miR-338-3p, miR-340-5p, miR-342-3p, miR-376a-3p, miR-486-5p, miR-652-3p	69.33
**miR-140-3p, miR-181a-5p, miR-192-5p, miR-22-3p, miR-29a-3p, miR-30b-5p, miR-338-3p, miR-340-5p, miR-342-3p, miR-486-5p, miR-652-3p**	**70.42**
miR-140-3p, miR-181a-5p, miR-22-3p, miR-26a-5p, miR-29a-3p, miR-30b-5p, miR-338-3p, miR-340-5p, miR-342-3p, miR-486-5p, miR-652-3p	69.49
miR-140-3p, miR-181a-5p, miR-22-3p, miR-29a-3p, miR-30b-5p, miR-335-5p, miR-338-3p, miR-340-5p, miR-342-3p, miR-486-5p, miR-652-3p	68.75
miR-140-3p, miR-181a-5p, miR-22-3p, miR-29a-3p, miR-30b-5p, miR-338-3p, miR-340-5p, miR-342-3p, miR-376a-3p, miR-486-5p, miR-652-3p	68.51
miR-140-3p, miR-181a-5p, miR-192-5p, miR-22-3p, miR-26a-5p, miR-29a-3p, miR-30b-5p, miR-338-3p, miR-340-5p, miR-342-3p, miR-486-5p, miR-652-3p	69.64
miR-140-3p, miR-181a-5p, miR-192-5p, miR-22-3p, miR-29a-3p, miR-30b-5p, miR-335-5p, miR-338-3p, miR-340-5p, miR-342-3p, miR-486-5p, miR-652-3p	68.81
miR-140-3p, miR-181a-5p, miR-192-5p, miR-22-3p, miR-29a-3p, miR-30b-5p, miR-338-3p, miR-340-5p, miR-342-3p, miR-376a-3p, miR-486-5p, miR-652-3p	69.28
miR-140-3p, miR-181a-5p, miR-192-5p, miR-22-3p, miR-26a-5p, miR-29a-3p, miR-30b-5p, miR-335-5p, miR-338-3p, miR-340-5p, miR-342-3p, miR-486-5p, miR-652-3p	67.83
miR-140-3p, miR-181a-5p, miR-192-5p, miR-22-3p, miR-26a-5p, miR-29a-3p, miR-30b-5p, miR-338-3p, miR-340-5p, miR-342-3p, miR-376a-3p, miR-486-5p, miR-652-3p	68.21
miR-140-3p, miR-181a-5p, miR-192-5p, miR-22-3p, miR-26a-5p, miR-29a-3p, miR-30b-5p, miR-335-5p, miR-338-3p, miR-340-5p, miR-342-3p, miR-376a-3p, miR-486-5p, miR-652-3p	67.83

The performance assessment of the various models was derived from internal validation, utilizing repeated cross-validation (5 repetitions, 5 folds).
