# Supplementary material for: Serum miRNA-based diagnostic models for endometriosis: from discovery to validation
Source: Hum Reprod. 2025 Nov 21;41(2):195–203. doi: 10.1093/humrep/deaf221 (PMC12864148; doi:10.1093/humrep/deaf221)
Supplement: deaf221_Supplementary_Table_S4 [file deaf221_supplementary_table_s4.pdf]

OXFORD
human reproduction
Human Reproduction, 2025, 1–2
https://doi.org/10.1093/humrep/deaf221
Supplementary Data

Supplementary Table S4. Diagnostic models built by the logistic regression (LR) algorithm to differentiate patients with deep infiltrating endometriosis (DIE) from controls (CTR).

LR models: DIE vs CTR	AUC
miR-140-3p	45.74
miR-181a-5p	64.97
miR-192-5p	48.13
miR-22-3p	50.01
miR-26a-5p	67.92
miR-29a-3p	62.34
miR-30b-5p	62.16
miR-335-5p	66.89
miR-338-3p	60.76
miR-340-5p	72.46
miR-342-3p	62.69
miR-376a-3p	63.92
miR-486-5p	64.94
miR-652-3p	65.66
miR-140-3p, miR-340-5p	72.22
miR-181a-5p, miR-340-5p	71.80
miR-192-5p, miR-340-5p	72.85
miR-22-3p, miR-340-5p	75.05
miR-26a-5p, miR-340-5p	72.07
miR-29a-3p, miR-340-5p	73.23
miR-30b-5p, miR-340-5p	73.71
miR-335-5p, miR-340-5p	72.33
miR-338-3p, miR-340-5p	74.25
miR-340-5p, miR-342-3p	73.27
miR-340-5p, miR-376a-3p	71.88
miR-340-5p, miR-486-5p	72.86
miR-340-5p, miR-652-3p	70.76
miR-140-3p, miR-22-3p, miR-340-5p	74.89
miR-181a-5p, miR-22-3p, miR-340-5p	74.86
miR-192-5p, miR-22-3p, miR-340-5p	74.98
miR-22-3p, miR-26a-5p, miR-340-5p	74.08
miR-22-3p, miR-29a-3p, miR-340-5p	74.60
miR-22-3p, miR-30b-5p, miR-340-5p	74.97
miR-22-3p, miR-335-5p, miR-340-5p	74.64
miR-22-3p, miR-338-3p, miR-340-5p	75.03
miR-22-3p, miR-340-5p, miR-342-3p	74.56
miR-22-3p, miR-340-5p, miR-376a-3p	74.54
miR-22-3p, miR-340-5p, miR-486-5p	74.99
miR-22-3p, miR-340-5p, miR-652-3p	77.16
miR-140-3p, miR-22-3p, miR-340-5p, miR-652-3p	76.72
miR-181a-5p, miR-22-3p, miR-340-5p, miR-652-3p	76.28
miR-192-5p, miR-22-3p, miR-340-5p, miR-652-3p	77.77
miR-22-3p, miR-26a-5p, miR-340-5p, miR-652-3p	76.31
miR-22-3p, miR-29a-3p, miR-340-5p, miR-652-3p	76.49
miR-22-3p, miR-30b-5p, miR-340-5p, miR-652-3p	76.66
miR-22-3p, miR-335-5p, miR-340-5p, miR-652-3p	76.77
miR-22-3p, miR-338-3p, miR-340-5p, miR-652-3p	76.45
miR-22-3p, miR-340-5p, miR-342-3p, miR-652-3p	76.62
miR-22-3p, miR-340-5p, miR-376a-3p, miR-652-3p	76.59
miR-22-3p, miR-340-5p, miR-486-5p, miR-652-3p	76.86
miR-140-3p, miR-192-5p, miR-22-3p, miR-340-5p, miR-652-3p	77.41
miR-181a-5p, miR-192-5p, miR-22-3p, miR-340-5p, miR-652-3p	77.09
miR-192-5p, miR-22-3p, miR-26a-5p, miR-340-5p, miR-652-3p	77.17
miR-192-5p, miR-22-3p, miR-29a-3p, miR-340-5p, miR-652-3p	77.19
miR-192-5p, miR-22-3p, miR-30b-5p, miR-340-5p, miR-652-3p	77.60
**miR-192-5p, miR-22-3p, miR-335-5p, miR-340-5p, miR-652-3p**	**77.90**
miR-192-5p, miR-22-3p, miR-338-3p, miR-340-5p, miR-652-3p	77.25
miR-192-5p, miR-22-3p, miR-340-5p, miR-342-3p, miR-652-3p	77.03
miR-192-5p, miR-22-3p, miR-340-5p, miR-376a-3p, miR-652-3p	77.51
miR-192-5p, miR-22-3p, miR-340-5p, miR-486-5p, miR-652-3p	77.37
miR-140-3p, miR-192-5p, miR-22-3p, miR-335-5p, miR-340-5p, miR-652-3p	77.77
miR-181a-5p, miR-192-5p, miR-22-3p, miR-335-5p, miR-340-5p, miR-652-3p	76.98

(continued)

Supplementary Table S4. (continued)

LR models: DIE vs CTR	AUC
miR-192-5p, miR-22-3p, miR-26a-5p, miR-335-5p, miR-340-5p, miR-652-3p	77.18
miR-192-5p, miR-22-3p, miR-29a-3p, miR-335-5p, miR-340-5p, miR-652-3p	77.43
miR-192-5p, miR-22-3p, miR-30b-5p, miR-335-5p, miR-340-5p, miR-652-3p	77.29
miR-192-5p, miR-22-3p, miR-335-5p, miR-338-3p, miR-340-5p, miR-652-3p	77.54
miR-192-5p, miR-22-3p, miR-335-5p, miR-340-5p, miR-342-3p, miR-652-3p	77.06
miR-192-5p, miR-22-3p, miR-335-5p, miR-340-5p, miR-376a-3p, miR-652-3p	77.36
miR-192-5p, miR-22-3p, miR-335-5p, miR-340-5p, miR-486-5p, miR-652-3p	77.21
miR-140-3p, miR-181a-5p, miR-192-5p, miR-22-3p, miR-335-5p, miR-340-5p, miR-652-3p	76.62
miR-140-3p, miR-192-5p, miR-22-3p, miR-26a-5p, miR-335-5p, miR-340-5p, miR-652-3p	77.25
miR-140-3p, miR-192-5p, miR-22-3p, miR-29a-3p, miR-335-5p, miR-340-5p, miR-652-3p	76.98
miR-140-3p, miR-192-5p, miR-22-3p, miR-30b-5p, miR-335-5p, miR-340-5p, miR-652-3p	77.09
miR-140-3p, miR-192-5p, miR-22-3p, miR-335-5p, miR-338-3p, miR-340-5p, miR-652-3p	77.12
miR-140-3p, miR-192-5p, miR-22-3p, miR-335-5p, miR-340-5p, miR-342-3p, miR-652-3p	76.86
miR-140-3p, miR-192-5p, miR-22-3p, miR-335-5p, miR-340-5p, miR-376a-3p, miR-652-3p	76.93
miR-140-3p, miR-192-5p, miR-22-3p, miR-335-5p, miR-340-5p, miR-486-5p, miR-652-3p	77.04
miR-140-3p, miR-181a-5p, miR-192-5p, miR-22-3p, miR-26a-5p, miR-335-5p, miR-340-5p, miR-652-3p	76.18
miR-140-3p, miR-192-5p, miR-22-3p, miR-26a-5p, miR-29a-3p, miR-335-5p, miR-340-5p, miR-652-3p	76.48
miR-140-3p, miR-192-5p, miR-22-3p, miR-26a-5p, miR-30b-5p, miR-335-5p, miR-340-5p, miR-652-3p	76.74
miR-140-3p, miR-192-5p, miR-22-3p, miR-26a-5p, miR-335-5p, miR-338-3p, miR-340-5p, miR-652-3p	76.50
miR-140-3p, miR-192-5p, miR-22-3p, miR-26a-5p, miR-335-5p, miR-340-5p, miR-342-3p, miR-652-3p	76.39
miR-140-3p, miR-192-5p, miR-22-3p, miR-26a-5p, miR-335-5p, miR-340-5p, miR-376a-3p, miR-652-3p	76.53
miR-140-3p, miR-192-5p, miR-22-3p, miR-26a-5p, miR-335-5p, miR-340-5p, miR-486-5p, miR-652-3p	76.80
miR-140-3p, miR-181a-5p, miR-192-5p, miR-22-3p, miR-26a-5p, miR-335-5p, miR-340-5p, miR-486-5p, miR-652-3p	75.73
miR-140-3p, miR-192-5p, miR-22-3p, miR-26a-5p, miR-29a-3p, miR-335-5p, miR-340-5p, miR-486-5p, miR-652-3p	75.82
miR-140-3p, miR-192-5p, miR-22-3p, miR-26a-5p, miR-30b-5p, miR-335-5p, miR-340-5p, miR-486-5p, miR-652-3p	76.41
miR-140-3p, miR-192-5p, miR-22-3p, miR-26a-5p, miR-335-5p, miR-338-3p, miR-340-5p, miR-486-5p, miR-652-3p	75.55
miR-140-3p, miR-192-5p, miR-22-3p, miR-26a-5p, miR-335-5p, miR-340-5p, miR-342-3p, miR-486-5p, miR-652-3p	75.92
miR-140-3p, miR-192-5p, miR-22-3p, miR-26a-5p, miR-335-5p, miR-340-5p, miR-376a-3p, miR-486-5p, miR-652-3p	76.11
miR-140-3p, miR-181a-5p, miR-192-5p, miR-22-3p, miR-26a-5p, miR-30b-5p, miR-335-5p, miR-340-5p, miR-486-5p, miR-652-3p	75.35
miR-140-3p, miR-192-5p, miR-22-3p, miR-26a-5p, miR-29a-3p, miR-30b-5p, miR-335-5p, miR-340-5p, miR-486-5p, miR-652-3p	75.50
miR-140-3p, miR-192-5p, miR-22-3p, miR-26a-5p, miR-30b-5p, miR-335-5p, miR-338-3p, miR-340-5p, miR-486-5p, miR-652-3p	75.41
miR-140-3p, miR-192-5p, miR-22-3p, miR-26a-5p, miR-30b-5p, miR-335-5p, miR-340-5p, miR-342-3p, miR-486-5p, miR-652-3p	75.63
miR-140-3p, miR-192-5p, miR-22-3p, miR-26a-5p, miR-30b-5p, miR-335-5p, miR-340-5p, miR-376a-3p, miR-486-5p, miR-652-3p	75.73
miR-140-3p, miR-181a-5p, miR-192-5p, miR-22-3p, miR-26a-5p, miR-30b-5p, miR-335-5p, miR-340-5p, miR-376a-3p, miR-486-5p, miR-652-3p	74.74
miR-140-3p, miR-192-5p, miR-22-3p, miR-26a-5p, miR-29a-3p, miR-30b-5p, miR-335-5p, miR-340-5p, miR-376a-3p, miR-486-5p, miR-652-3p	74.88
miR-140-3p, miR-192-5p, miR-22-3p, miR-26a-5p, miR-30b-5p, miR-335-5p, miR-338-3p, miR-340-5p, miR-376a-3p, miR-486-5p, miR-652-3p	74.90
miR-140-3p, miR-192-5p, miR-22-3p, miR-26a-5p, miR-30b-5p, miR-335-5p, miR-340-5p, miR-342-3p, miR-376a-3p, miR-486-5p, miR-652-3p	74.96
miR-140-3p, miR-181a-5p, miR-192-5p, miR-22-3p, miR-26a-5p, miR-30b-5p, miR-335-5p, miR-340-5p, miR-342-3p, miR-376a-3p, miR-486-5p, miR-652-3p	74.03
miR-140-3p, miR-192-5p, miR-22-3p, miR-26a-5p, miR-29a-3p, miR-30b-5p, miR-335-5p, miR-340-5p, miR-342-3p, miR-376a-3p, miR-486-5p, miR-652-3p	74.66
miR-140-3p, miR-192-5p, miR-22-3p, miR-26a-5p, miR-30b-5p, miR-335-5p, miR-338-3p, miR-340-5p, miR-342-3p, miR-376a-3p, miR-486-5p, miR-652-3p	74.42
miR-140-3p, miR-181a-5p, miR-192-5p, miR-22-3p, miR-26a-5p, miR-29a-3p, miR-30b-5p, miR-335-5p, miR-340-5p, miR-342-3p, miR-376a-3p, miR-486-5p, miR-652-3p	73.68
miR-140-3p, miR-192-5p, miR-22-3p, miR-26a-5p, miR-29a-3p, miR-30b-5p, miR-335-5p, miR-338-3p, miR-340-5p, miR-342-3p, miR-376a-3p, miR-486-5p, miR-652-3p	73.89
miR-140-3p, miR-181a-5p, miR-192-5p, miR-22-3p, miR-26a-5p, miR-29a-3p, miR-30b-5p, miR-335-5p, miR-338-3p, miR-340-5p, miR-342-3p, miR-376a-3p, miR-486-5p, miR-652-3p	73.11

The performance assessment of the various models was derived from internal validation, utilizing repeated cross-validation (5 repetitions, 5 folds).
