# Supplementary material for: Serum miRNA-based diagnostic models for endometriosis: from discovery to validation
Source: Hum Reprod. 2025 Nov 21;41(2):195–203. doi: 10.1093/humrep/deaf221 (PMC12864148; doi:10.1093/humrep/deaf221)
Supplement: deaf221_Supplementary_Table_S5 [file deaf221_supplementary_table_s5.pdf]

OXFORD

human reproduction

Human Reproduction, 2025, 1–2

https://doi.org/10.1093/humrep/deaf221

Supplementary Data

Supplementary Table S5. Diagnostic models built by the Random Forest (RF) algorithm to differentiate patients with deep infiltrating endometriosis (DIE) from controls (CTR).

RF models: DIE vs CTR	AUC
miR-140-3p	58.32
miR-181a-5p	53.71
miR-192-5p	53.34
miR-22-3p	54.56
miR-26a-5p	58.10
miR-29a-3p	57.18
miR-30b-5p	58.61
miR-335-5p	55.64
miR-338-3p	50.45
miR-340-5p	65.14
miR-342-3p	55.29
miR-376a-3p	53.37
miR-486-5p	65.64
miR-652-3p	59.17
miR-140-3p, miR-486-5p	68.61
miR-181a-5p, miR-486-5p	68.26
miR-192-5p, miR-486-5p	71.69
miR-22-3p, miR-486-5p	68.85
miR-26a-5p, miR-486-5p	66.54
miR-29a-3p, miR-486-5p	63.83
miR-30b-5p, miR-486-5p	70.18
miR-335-5p, miR-486-5p	71.07
miR-338-3p, miR-486-5p	68.88
miR-340-5p, miR-486-5p	65.01
miR-342-3p, miR-486-5p	67.70
miR-376a-3p, miR-486-5p	69.82
miR-486-5p, miR-652-3p	71.95
miR-140-3p, miR-486-5p, miR-652-3p	73.55
miR-181a-5p, miR-486-5p, miR-652-3p	72.83
miR-192-5p, miR-486-5p, miR-652-3p	75.47
miR-22-3p, miR-486-5p, miR-652-3p	74.24
miR-26a-5p, miR-486-5p, miR-652-3p	73.68
miR-29a-3p, miR-486-5p, miR-652-3p	71.07
miR-30b-5p, miR-486-5p, miR-652-3p	76.70
miR-335-5p, miR-486-5p, miR-652-3p	76.06
miR-338-3p, miR-486-5p, miR-652-3p	76.65
miR-340-5p, miR-486-5p, miR-652-3p	72.12
miR-342-3p, miR-486-5p, miR-652-3p	72.18
miR-376a-3p, miR-486-5p, miR-652-3p	74.91
miR-140-3p, miR-30b-5p, miR-486-5p, miR-652-3p	76.40
miR-181a-5p, miR-30b-5p, miR-486-5p, miR-652-3p	74.45
miR-192-5p, miR-30b-5p, miR-486-5p, miR-652-3p	77.18
miR-22-3p, miR-30b-5p, miR-486-5p, miR-652-3p	74.55
miR-26a-5p, miR-30b-5p, miR-486-5p, miR-652-3p	76.24
miR-29a-3p, miR-30b-5p, miR-486-5p, miR-652-3p	75.03
miR-30b-5p, miR-335-5p, miR-486-5p, miR-652-3p	76.41
miR-30b-5p, miR-338-3p, miR-486-5p, miR-652-3p	77.96
miR-30b-5p, miR-340-5p, miR-486-5p, miR-652-3p	75.27
miR-30b-5p, miR-342-3p, miR-486-5p, miR-652-3p	75.71
miR-30b-5p, miR-376a-3p, miR-486-5p, miR-652-3p	74.93
miR-140-3p, miR-30b-5p, miR-338-3p, miR-486-5p, miR-652-3p	78.61
miR-181a-5p, miR-30b-5p, miR-338-3p, miR-486-5p, miR-652-3p	77.28
miR-192-5p, miR-30b-5p, miR-338-3p, miR-486-5p, miR-652-3p	79.00
miR-22-3p, miR-30b-5p, miR-338-3p, miR-486-5p, miR-652-3p	76.82
miR-26a-5p, miR-30b-5p, miR-338-3p, miR-486-5p, miR-652-3p	75.98
miR-29a-3p, miR-30b-5p, miR-338-3p, miR-486-5p, miR-652-3p	77.10
miR-30b-5p, miR-335-5p, miR-338-3p, miR-486-5p, miR-652-3p	78.57
miR-30b-5p, miR-338-3p, miR-340-5p, miR-486-5p, miR-652-3p	75.84
miR-30b-5p, miR-338-3p, miR-342-3p, miR-486-5p, miR-652-3p	75.47
miR-30b-5p, miR-338-3p, miR-376a-3p, miR-486-5p, miR-652-3p	76.38
miR-140-3p, miR-192-5p, miR-30b-5p, miR-338-3p, miR-486-5p, miR-652-3p	78.93
miR-181a-5p, miR-192-5p, miR-30b-5p, miR-338-3p, miR-486-5p, miR-652-3p	77.37

(continued)

Supplementary Table S5. (continued)

RF models: DIE vs CTR	AUC
miR-192-5p, miR-22-3p, miR-30b-5p, miR-338-3p, miR-486-5p, miR-652-3p	78.32
miR-192-5p, miR-26a-5p, miR-30b-5p, miR-338-3p, miR-486-5p, miR-652-3p	77.08
miR-192-5p, miR-29a-3p, miR-30b-5p, miR-338-3p, miR-486-5p, miR-652-3p	77.50
**miR-192-5p, miR-30b-5p, miR-335-5p, miR-338-3p, miR-486-5p, miR-652-3p**	**80.36**
miR-192-5p, miR-30b-5p, miR-338-3p, miR-340-5p, miR-486-5p, miR-652-3p	77.53
miR-192-5p, miR-30b-5p, miR-338-3p, miR-342-3p, miR-486-5p, miR-652-3p	76.12
miR-192-5p, miR-30b-5p, miR-338-3p, miR-376a-3p, miR-486-5p, miR-652-3p	77.90
miR-140-3p, miR-192-5p, miR-30b-5p, miR-335-5p, miR-338-3p, miR-486-5p, miR-652-3p	80.08
miR-181a-5p, miR-192-5p, miR-30b-5p, miR-335-5p, miR-338-3p, miR-486-5p, miR-652-3p	78.53
miR-192-5p, miR-22-3p, miR-30b-5p, miR-335-5p, miR-338-3p, miR-486-5p, miR-652-3p	78.62
miR-192-5p, miR-26a-5p, miR-30b-5p, miR-335-5p, miR-338-3p, miR-486-5p, miR-652-3p	78.52
miR-192-5p, miR-29a-3p, miR-30b-5p, miR-335-5p, miR-338-3p, miR-486-5p, miR-652-3p	79.80
miR-192-5p, miR-30b-5p, miR-335-5p, miR-338-3p, miR-340-5p, miR-486-5p, miR-652-3p	79.29
miR-192-5p, miR-30b-5p, miR-335-5p, miR-338-3p, miR-342-3p, miR-486-5p, miR-652-3p	77.67
miR-192-5p, miR-30b-5p, miR-335-5p, miR-338-3p, miR-376a-3p, miR-486-5p, miR-652-3p	80.14
miR-140-3p, miR-192-5p, miR-30b-5p, miR-335-5p, miR-338-3p, miR-376a-3p, miR-486-5p, miR-652-3p	79.54
miR-181a-5p, miR-192-5p, miR-30b-5p, miR-335-5p, miR-338-3p, miR-376a-3p, miR-486-5p, miR-652-3p	78.43
miR-192-5p, miR-22-3p, miR-30b-5p, miR-335-5p, miR-338-3p, miR-376a-3p, miR-486-5p, miR-652-3p	79.14
miR-192-5p, miR-26a-5p, miR-30b-5p, miR-335-5p, miR-338-3p, miR-376a-3p, miR-486-5p, miR-652-3p	78.12
miR-192-5p, miR-29a-3p, miR-30b-5p, miR-335-5p, miR-338-3p, miR-376a-3p, miR-486-5p, miR-652-3p	78.50
miR-192-5p, miR-30b-5p, miR-335-5p, miR-338-3p, miR-340-5p, miR-376a-3p, miR-486-5p, miR-652-3p	78.75
miR-192-5p, miR-30b-5p, miR-335-5p, miR-338-3p, miR-342-3p, miR-376a-3p, miR-486-5p, miR-652-3p	76.98
miR-140-3p, miR-181a-5p, miR-192-5p, miR-30b-5p, miR-335-5p, miR-338-3p, miR-376a-3p, miR-486-5p, miR-652-3p	78.77
miR-140-3p, miR-192-5p, miR-22-3p, miR-30b-5p, miR-335-5p, miR-338-3p, miR-376a-3p, miR-486-5p, miR-652-3p	78.28
miR-140-3p, miR-192-5p, miR-26a-5p, miR-30b-5p, miR-335-5p, miR-338-3p, miR-376a-3p, miR-486-5p, miR-652-3p	78.07
miR-140-3p, miR-192-5p, miR-29a-3p, miR-30b-5p, miR-335-5p, miR-338-3p, miR-376a-3p, miR-486-5p, miR-652-3p	78.51
miR-140-3p, miR-192-5p, miR-30b-5p, miR-335-5p, miR-338-3p, miR-340-5p, miR-376a-3p, miR-486-5p, miR-652-3p	79.36
miR-140-3p, miR-192-5p, miR-30b-5p, miR-335-5p, miR-338-3p, miR-342-3p, miR-376a-3p, miR-486-5p, miR-652-3p	77.86
miR-140-3p, miR-181a-5p, miR-192-5p, miR-30b-5p, miR-335-5p, miR-338-3p, miR-340-5p, miR-376a-3p, miR-486-5p, miR-652-3p	78.32
miR-140-3p, miR-192-5p, miR-22-3p, miR-30b-5p, miR-335-5p, miR-338-3p, miR-340-5p, miR-376a-3p, miR-486-5p, miR-652-3p	79.14
miR-140-3p, miR-192-5p, miR-26a-5p, miR-30b-5p, miR-335-5p, miR-338-3p, miR-340-5p, miR-376a-3p, miR-486-5p, miR-652-3p	77.65
miR-140-3p, miR-192-5p, miR-29a-3p, miR-30b-5p, miR-335-5p, miR-338-3p, miR-340-5p, miR-376a-3p, miR-486-5p, miR-652-3p	78.50
miR-140-3p, miR-192-5p, miR-30b-5p, miR-335-5p, miR-338-3p, miR-340-5p, miR-342-3p, miR-376a-3p, miR-486-5p, miR-652-3p	76.60
miR-140-3p, miR-181a-5p, miR-192-5p, miR-22-3p, miR-30b-5p, miR-335-5p, miR-338-3p, miR-340-5p, miR-376a-3p, miR-486-5p, miR-652-3p	78.46
miR-140-3p, miR-192-5p, miR-22-3p, miR-26a-5p, miR-30b-5p, miR-335-5p, miR-338-3p, miR-340-5p, miR-376a-3p, miR-486-5p, miR-652-3p	77.86
miR-140-3p, miR-192-5p, miR-22-3p, miR-29a-3p, miR-30b-5p, miR-335-5p, miR-338-3p, miR-340-5p, miR-376a-3p, miR-486-5p, miR-652-3p	78.44
miR-140-3p, miR-192-5p, miR-22-3p, miR-30b-5p, miR-335-5p, miR-338-3p, miR-340-5p, miR-342-3p, miR-376a-3p, miR-486-5p, miR-652-3p	77.22
miR-140-3p, miR-181a-5p, miR-192-5p, miR-22-3p, miR-26a-5p, miR-30b-5p, miR-335-5p, miR-338-3p, miR-340-5p, miR-376a-3p, miR-486-5p, miR-652-3p	76.75
miR-140-3p, miR-181a-5p, miR-192-5p, miR-22-3p, miR-29a-3p, miR-30b-5p, miR-335-5p, miR-338-3p, miR-340-5p, miR-376a-3p, miR-486-5p, miR-652-3p	77.48
miR-140-3p, miR-181a-5p, miR-192-5p, miR-22-3p, miR-30b-5p, miR-335-5p, miR-338-3p, miR-340-5p, miR-342-3p, miR-376a-3p, miR-486-5p, miR-652-3p	77.32
miR-140-3p, miR-181a-5p, miR-192-5p, miR-22-3p, miR-26a-5p, miR-29a-3p, miR-30b-5p, miR-335-5p, miR-338-3p, miR-340-5p, miR-376a-3p, miR-486-5p, miR-652-3p	76.87
miR-140-3p, miR-181a-5p, miR-192-5p, miR-22-3p, miR-29a-3p, miR-30b-5p, miR-335-5p, miR-338-3p, miR-340-5p, miR-342-3p, miR-376a-3p, miR-486-5p, miR-652-3p	76.27
miR-140-3p, miR-181a-5p, miR-192-5p, miR-22-3p, miR-26a-5p, miR-29a-3p, miR-30b-5p, miR-335-5p, miR-338-3p, miR-340-5p, miR-342-3p, miR-376a-3p, miR-486-5p, miR-652-3p	76.15

The performance assessment of the various models was derived from internal validation, utilizing repeated cross-validation (5 repetitions, 5 folds).
