# Supplementary material for: Serum miRNA-based diagnostic models for endometriosis: from discovery to validation
Source: Hum Reprod. 2025 Nov 21;41(2):195–203. doi: 10.1093/humrep/deaf221 (PMC12864148; doi:10.1093/humrep/deaf221)
Supplement: deaf221_Supplementary_Table_S6 [file deaf221_supplementary_table_s6.pdf]

OXFORD
human reproduction
Human Reproduction, 2025, 1–2
https://doi.org/10.1093/humrep/deaf221
Supplementary Data

Supplementary Table S6. Diagnostic models built by the logistic regression (LR) algorithm to differentiate patients with ovarian endometrioma (OMA) from controls (CTR).

LR models: OMA vs CTR	AUC
miR-140-3p	58.25
miR-181a-5p	60.83
miR-192-5p	58.32
miR-22-3p	57.62
miR-26a-5p	61.26
miR-29a-3p	46.26
miR-30b-5p	58.92
miR-335-5p	57.98
miR-338-3p	46.66
miR-340-5p	44.57
miR-342-3p	62.95
miR-376a-3p	46.22
miR-486-5p	43.91
miR-652-3p	45.69
miR-140-3p, miR-342-3p	63.77
**miR-181a-5p, miR-342-3p**	**65.39**
miR-192-5p, miR-342-3p	62.25
miR-22-3p, miR-342-3p	61.66
miR-26a-5p, miR-342-3p	63.17
miR-29a-3p, miR-342-3p	62.23
miR-30b-5p, miR-342-3p	61.49
miR-335-5p, miR-342-3p	61.76
miR-338-3p, miR-342-3p	62.16
miR-340-5p, miR-342-3p	61.68
miR-342-3p, miR-376a-3p	62.22
miR-342-3p, miR-486-5p	62.73
miR-342-3p, miR-652-3p	60.73
miR-140-3p, miR-181a-5p, miR-342-3p	65.09
miR-181a-5p, miR-192-5p, miR-342-3p	64.35
miR-181a-5p, miR-22-3p, miR-342-3p	64.36
miR-181a-5p, miR-26a-5p, miR-342-3p	63.47
miR-181a-5p, miR-29a-3p, miR-342-3p	64.64
miR-181a-5p, miR-30b-5p, miR-342-3p	63.42
miR-181a-5p, miR-335-5p, miR-342-3p	63.93
miR-181a-5p, miR-338-3p, miR-342-3p	64.25
miR-181a-5p, miR-340-5p, miR-342-3p	63.85
miR-181a-5p, miR-342-3p, miR-376a-3p	64.50
miR-181a-5p, miR-342-3p, miR-486-5p	64.28
miR-181a-5p, miR-342-3p, miR-652-3p	63.46
miR-140-3p, miR-181a-5p, miR-192-5p, miR-342-3p	64.35
miR-140-3p, miR-181a-5p, miR-22-3p, miR-342-3p	64.27
miR-140-3p, miR-181a-5p, miR-26a-5p, miR-342-3p	63.03
miR-140-3p, miR-181a-5p, miR-29a-3p, miR-342-3p	64.84
miR-140-3p, miR-181a-5p, miR-30b-5p, miR-342-3p	63.65
miR-140-3p, miR-181a-5p, miR-335-5p, miR-342-3p	64.08
miR-140-3p, miR-181a-5p, miR-338-3p, miR-342-3p	64.86
miR-140-3p, miR-181a-5p, miR-340-5p, miR-342-3p	64.32
miR-140-3p, miR-181a-5p, miR-342-3p, miR-376a-3p	64.85
miR-140-3p, miR-181a-5p, miR-342-3p, miR-486-5p	64.32
miR-140-3p, miR-181a-5p, miR-342-3p, miR-652-3p	63.67
miR-140-3p, miR-181a-5p, miR-192-5p, miR-338-3p, miR-342-3p	63.96
miR-140-3p, miR-181a-5p, miR-22-3p, miR-338-3p, miR-342-3p	63.43
miR-140-3p, miR-181a-5p, miR-26a-5p, miR-338-3p, miR-342-3p	62.97
miR-140-3p, miR-181a-5p, miR-29a-3p, miR-338-3p, miR-342-3p	64.12
miR-140-3p, miR-181a-5p, miR-30b-5p, miR-338-3p, miR-342-3p	63.93
miR-140-3p, miR-181a-5p, miR-335-5p, miR-338-3p, miR-342-3p	63.70
miR-140-3p, miR-181a-5p, miR-338-3p, miR-340-5p, miR-342-3p	63.83
miR-140-3p, miR-181a-5p, miR-338-3p, miR-342-3p, miR-376a-3p	64.11
miR-140-3p, miR-181a-5p, miR-338-3p, miR-342-3p, miR-486-5p	64.54
miR-140-3p, miR-181a-5p, miR-338-3p, miR-342-3p, miR-652-3p	64.46
miR-140-3p, miR-181a-5p, miR-192-5p, miR-338-3p, miR-342-3p, miR-486-5p	64.46
miR-140-3p, miR-181a-5p, miR-22-3p, miR-338-3p, miR-342-3p, miR-486-5p	63.41

(continued)

Supplementary Table S6. (continued)

LR models: OMA vs CTR	AUC
miR-140-3p, miR-181a-5p, miR-26a-5p, miR-338-3p, miR-342-3p, miR-486-5p	63.09
miR-140-3p, miR-181a-5p, miR-29a-3p, miR-338-3p, miR-342-3p, miR-486-5p	63.92
miR-140-3p, miR-181a-5p, miR-30b-5p, miR-338-3p, miR-342-3p, miR-486-5p	63.74
miR-140-3p, miR-181a-5p, miR-335-5p, miR-338-3p, miR-342-3p, miR-486-5p	63.61
miR-140-3p, miR-181a-5p, miR-338-3p, miR-340-5p, miR-342-3p, miR-486-5p	63.46
miR-140-3p, miR-181a-5p, miR-338-3p, miR-342-3p, miR-376a-3p, miR-486-5p	64.17
miR-140-3p, miR-181a-5p, miR-338-3p, miR-342-3p, miR-486-5p, miR-652-3p	64.04
miR-140-3p, miR-181a-5p, miR-192-5p, miR-22-3p, miR-338-3p, miR-342-3p, miR-486-5p	63.63
miR-140-3p, miR-181a-5p, miR-192-5p, miR-26a-5p, miR-338-3p, miR-342-3p, miR-486-5p	62.88
miR-140-3p, miR-181a-5p, miR-192-5p, miR-29a-3p, miR-338-3p, miR-342-3p, miR-486-5p	63.81
miR-140-3p, miR-181a-5p, miR-192-5p, miR-30b-5p, miR-338-3p, miR-342-3p, miR-486-5p	63.68
miR-140-3p, miR-181a-5p, miR-192-5p, miR-335-5p, miR-338-3p, miR-342-3p, miR-486-5p	63.38
miR-140-3p, miR-181a-5p, miR-192-5p, miR-338-3p, miR-340-5p, miR-342-3p, miR-486-5p	63.53
miR-140-3p, miR-181a-5p, miR-192-5p, miR-338-3p, miR-342-3p, miR-376a-3p, miR-486-5p	63.84
miR-140-3p, miR-181a-5p, miR-192-5p, miR-338-3p, miR-342-3p, miR-486-5p, miR-652-3p	63.79
miR-140-3p, miR-181a-5p, miR-192-5p, miR-22-3p, miR-338-3p, miR-342-3p, miR-376a-3p, miR-486-5p	63.33
miR-140-3p, miR-181a-5p, miR-192-5p, miR-26a-5p, miR-338-3p, miR-342-3p, miR-376a-3p, miR-486-5p	62.43
miR-140-3p, miR-181a-5p, miR-192-5p, miR-29a-3p, miR-338-3p, miR-342-3p, miR-376a-3p, miR-486-5p	63.16
miR-140-3p, miR-181a-5p, miR-192-5p, miR-30b-5p, miR-338-3p, miR-342-3p, miR-376a-3p, miR-486-5p	63.20
miR-140-3p, miR-181a-5p, miR-192-5p, miR-335-5p, miR-338-3p, miR-342-3p, miR-376a-3p, miR-486-5p	62.82
miR-140-3p, miR-181a-5p, miR-192-5p, miR-338-3p, miR-340-5p, miR-342-3p, miR-376a-3p, miR-486-5p	62.95
miR-140-3p, miR-181a-5p, miR-192-5p, miR-338-3p, miR-342-3p, miR-376a-3p, miR-486-5p, miR-652-3p	63.52
miR-140-3p, miR-181a-5p, miR-192-5p, miR-22-3p, miR-338-3p, miR-342-3p, miR-376a-3p, miR-486-5p, miR-652-3p	64.14
miR-140-3p, miR-181a-5p, miR-192-5p, miR-26a-5p, miR-338-3p, miR-342-3p, miR-376a-3p, miR-486-5p, miR-652-3p	62.16
miR-140-3p, miR-181a-5p, miR-192-5p, miR-29a-3p, miR-338-3p, miR-342-3p, miR-376a-3p, miR-486-5p, miR-652-3p	62.98
miR-140-3p, miR-181a-5p, miR-192-5p, miR-30b-5p, miR-338-3p, miR-342-3p, miR-376a-3p, miR-486-5p, miR-652-3p	63.12
miR-140-3p, miR-181a-5p, miR-192-5p, miR-335-5p, miR-338-3p, miR-342-3p, miR-376a-3p, miR-486-5p, miR-652-3p	62.68
miR-140-3p, miR-181a-5p, miR-192-5p, miR-338-3p, miR-340-5p, miR-342-3p, miR-376a-3p, miR-486-5p, miR-652-3p	62.85
miR-140-3p, miR-181a-5p, miR-192-5p, miR-22-3p, miR-26a-5p, miR-338-3p, miR-342-3p, miR-376a-3p, miR-486-5p, miR-652-3p	62.80
miR-140-3p, miR-181a-5p, miR-192-5p, miR-22-3p, miR-29a-3p, miR-338-3p, miR-342-3p, miR-376a-3p, miR-486-5p, miR-652-3p	63.43
miR-140-3p, miR-181a-5p, miR-192-5p, miR-22-3p, miR-30b-5p, miR-338-3p, miR-342-3p, miR-376a-3p, miR-486-5p, miR-652-3p	63.86
miR-140-3p, miR-181a-5p, miR-192-5p, miR-22-3p, miR-335-5p, miR-338-3p, miR-342-3p, miR-376a-3p, miR-486-5p, miR-652-3p	63.17
miR-140-3p, miR-181a-5p, miR-192-5p, miR-22-3p, miR-338-3p, miR-340-5p, miR-342-3p, miR-376a-3p, miR-486-5p, miR-652-3p	63.34
miR-140-3p, miR-181a-5p, miR-192-5p, miR-22-3p, miR-26a-5p, miR-30b-5p, miR-338-3p, miR-342-3p, miR-376a-3p, miR-486-5p, miR-652-3p	63.00
miR-140-3p, miR-181a-5p, miR-192-5p, miR-22-3p, miR-29a-3p, miR-30b-5p, miR-338-3p, miR-342-3p, miR-376a-3p, miR-486-5p, miR-652-3p	63.30
miR-140-3p, miR-181a-5p, miR-192-5p, miR-22-3p, miR-30b-5p, miR-335-5p, miR-338-3p, miR-342-3p, miR-376a-3p, miR-486-5p, miR-652-3p	63.29
miR-140-3p, miR-181a-5p, miR-192-5p, miR-22-3p, miR-30b-5p, miR-338-3p, miR-340-5p, miR-342-3p, miR-376a-3p, miR-486-5p, miR-652-3p	63.28
miR-140-3p, miR-181a-5p, miR-192-5p, miR-22-3p, miR-26a-5p, miR-29a-3p, miR-30b-5p, miR-338-3p, miR-342-3p, miR-376a-3p, miR-486-5p, miR-652-3p	62.36
miR-140-3p, miR-181a-5p, miR-192-5p, miR-22-3p, miR-29a-3p, miR-30b-5p, miR-335-5p, miR-338-3p, miR-342-3p, miR-376a-3p, miR-486-5p, miR-652-3p	62.76
miR-140-3p, miR-181a-5p, miR-192-5p, miR-22-3p, miR-29a-3p, miR-30b-5p, miR-338-3p, miR-340-5p, miR-342-3p, miR-376a-3p, miR-486-5p, miR-652-3p	62.70
miR-140-3p, miR-181a-5p, miR-192-5p, miR-22-3p, miR-26a-5p, miR-29a-3p, miR-30b-5p, miR-335-5p, miR-338-3p, miR-342-3p, miR-376a-3p, miR-486-5p, miR-652-3p	61.73
miR-140-3p, miR-181a-5p, miR-192-5p, miR-22-3p, miR-29a-3p, miR-30b-5p, miR-335-5p, miR-338-3p, miR-340-5p, miR-342-3p, miR-376a-3p, miR-486-5p, miR-652-3p	62.12
miR-140-3p, miR-181a-5p, miR-192-5p, miR-22-3p, miR-26a-5p, miR-29a-3p, miR-30b-5p, miR-335-5p, miR-338-3p, miR-340-5p, miR-342-3p, miR-376a-3p, miR-486-5p, miR-652-3p	60.99

The performance assessment of the various models was derived from internal validation, utilizing repeated cross-validation (5 repetitions, 5 folds).
