# Supplementary material for: Serum miRNA-based diagnostic models for endometriosis: from discovery to validation
Source: Hum Reprod. 2025 Nov 21;41(2):195–203. doi: 10.1093/humrep/deaf221 (PMC12864148; doi:10.1093/humrep/deaf221)
Supplement: deaf221_Supplementary_Table_S7 [file deaf221_supplementary_table_s7.pdf]

OXFORD
human reproduction
Human Reproduction, 2025, 1–2
https://doi.org/10.1093/humrep/deaf221
Supplementary Data

Supplementary Table S7. Diagnostic models built by the Random Forest (RF) algorithm to differentiate patients with ovarian endometrioma (OMA) from controls (CTR).

RF models: OMA vs CTR	AUC
miR-140-3p	50.44
miR-181a-5p	50.27
miR-192-5p	51.22
miR-22-3p	47.14
miR-26a-5p	57.01
miR-29a-3p	53.73
miR-30b-5p	51.95
miR-335-5p	44.57
miR-338-3p	50.24
miR-340-5p	46.15
miR-342-3p	52.84
miR-376a-3p	41.98
miR-486-5p	54.82
miR-652-3p	50.40
miR-140-3p, miR-26a-5p	57.03
miR-181a-5p, miR-26a-5p	55.55
miR-192-5p, miR-26a-5p	55.05
miR-22-3p, miR-26a-5p	49.91
miR-26a-5p, miR-29a-3p	56.30
miR-26a-5p, miR-30b-5p	60.78
miR-26a-5p, miR-335-5p	54.16
miR-26a-5p, miR-338-3p	60.19
miR-26a-5p, miR-340-5p	52.96
miR-26a-5p, miR-342-3p	58.74
miR-26a-5p, miR-376a-3p	49.12
miR-26a-5p, miR-486-5p	62.24
miR-26a-5p, miR-652-3p	62.11
miR-140-3p, miR-26a-5p, miR-486-5p	59.63
miR-181a-5p, miR-26a-5p, miR-486-5p	61.46
miR-192-5p, miR-26a-5p, miR-486-5p	60.11
miR-22-3p, miR-26a-5p, miR-486-5p	61.34
miR-26a-5p, miR-29a-3p, miR-486-5p	61.03
miR-26a-5p, miR-30b-5p, miR-486-5p	58.90
miR-26a-5p, miR-335-5p, miR-486-5p	58.85
miR-26a-5p, miR-338-3p, miR-486-5p	62.16
miR-26a-5p, miR-340-5p, miR-486-5p	59.99
miR-26a-5p, miR-342-3p, miR-486-5p	62.09
miR-26a-5p, miR-376a-3p, miR-486-5p	51.69
miR-26a-5p, miR-486-5p, miR-652-3p	62.00
miR-140-3p, miR-26a-5p, miR-338-3p, miR-486-5p	60.83
miR-181a-5p, miR-26a-5p, miR-338-3p, miR-486-5p	61.23
miR-192-5p, miR-26a-5p, miR-338-3p, miR-486-5p	59.98
miR-22-3p, miR-26a-5p, miR-338-3p, miR-486-5p	62.21
miR-26a-5p, miR-29a-3p, miR-338-3p, miR-486-5p	59.93
miR-26a-5p, miR-30b-5p, miR-338-3p, miR-486-5p	59.49
miR-26a-5p, miR-335-5p, miR-338-3p, miR-486-5p	60.47
miR-26a-5p, miR-338-3p, miR-340-5p, miR-486-5p	62.12
miR-26a-5p, miR-338-3p, miR-342-3p, miR-486-5p	62.12
miR-26a-5p, miR-338-3p, miR-376a-3p, miR-486-5p	51.88
miR-26a-5p, miR-338-3p, miR-486-5p, miR-652-3p	62.23
miR-140-3p, miR-26a-5p, miR-338-3p, miR-486-5p, miR-652-3p	61.82
miR-181a-5p, miR-26a-5p, miR-338-3p, miR-486-5p, miR-652-3p	58.35
miR-192-5p, miR-26a-5p, miR-338-3p, miR-486-5p, miR-652-3p	61.24
miR-22-3p, miR-26a-5p, miR-338-3p, miR-486-5p, miR-652-3p	61.59
miR-26a-5p, miR-29a-3p, miR-338-3p, miR-486-5p, miR-652-3p	58.42
miR-26a-5p, miR-30b-5p, miR-338-3p, miR-486-5p, miR-652-3p	59.65
miR-26a-5p, miR-335-5p, miR-338-3p, miR-486-5p, miR-652-3p	61.84
miR-26a-5p, miR-338-3p, miR-340-5p, miR-486-5p, miR-652-3p	60.33
miR-26a-5p, miR-338-3p, miR-342-3p, miR-486-5p, miR-652-3p	63.10
miR-26a-5p, miR-338-3p, miR-376a-3p, miR-486-5p, miR-652-3p	53.84
miR-140-3p, miR-26a-5p, miR-338-3p, miR-342-3p, miR-486-5p, miR-652-3p	65.43
miR-181a-5p, miR-26a-5p, miR-338-3p, miR-342-3p, miR-486-5p, miR-652-3p	60.36

(continued)

Supplementary Table S7. (continued)

RF models: OMA vs CTR	AUC
miR-192-5p, miR-26a-5p, miR-338-3p, miR-342-3p, miR-486-5p, miR-652-3p	61.15
miR-22-3p, miR-26a-5p, miR-338-3p, miR-342-3p, miR-486-5p, miR-652-3p	63.75
miR-26a-5p, miR-29a-3p, miR-338-3p, miR-342-3p, miR-486-5p, miR-652-3p	60.55
miR-26a-5p, miR-30b-5p, miR-338-3p, miR-342-3p, miR-486-5p, miR-652-3p	63.05
miR-26a-5p, miR-335-5p, miR-338-3p, miR-342-3p, miR-486-5p, miR-652-3p	62.34
miR-26a-5p, miR-338-3p, miR-340-5p, miR-342-3p, miR-486-5p, miR-652-3p	61.31
miR-26a-5p, miR-338-3p, miR-342-3p, miR-376a-3p, miR-486-5p, miR-652-3p	56.97
miR-140-3p, miR-181a-5p, miR-26a-5p, miR-338-3p, miR-342-3p, miR-486-5p, miR-652-3p	60.93
miR-140-3p, miR-192-5p, miR-26a-5p, miR-338-3p, miR-342-3p, miR-486-5p, miR-652-3p	64.22
miR-140-3p, miR-22-3p, miR-26a-5p, miR-338-3p, miR-342-3p, miR-486-5p, miR-652-3p	65.40
miR-140-3p, miR-26a-5p, miR-29a-3p, miR-338-3p, miR-342-3p, miR-486-5p, miR-652-3p	62.56
**miR-140-3p, miR-26a-5p, miR-30b-5p, miR-338-3p, miR-342-3p, miR-486-5p, miR-652-3p**	**65.84**
miR-140-3p, miR-26a-5p, miR-335-5p, miR-338-3p, miR-342-3p, miR-486-5p, miR-652-3p	64.56
miR-140-3p, miR-26a-5p, miR-338-3p, miR-340-5p, miR-342-3p, miR-486-5p, miR-652-3p	63.51
miR-140-3p, miR-26a-5p, miR-338-3p, miR-342-3p, miR-376a-3p, miR-486-5p, miR-652-3p	59.65
miR-140-3p, miR-181a-5p, miR-26a-5p, miR-30b-5p, miR-338-3p, miR-342-3p, miR-486-5p, miR-652-3p	61.99
miR-140-3p, miR-192-5p, miR-26a-5p, miR-30b-5p, miR-338-3p, miR-342-3p, miR-486-5p, miR-652-3p	64.46
miR-140-3p, miR-22-3p, miR-26a-5p, miR-30b-5p, miR-338-3p, miR-342-3p, miR-486-5p, miR-652-3p	64.51
miR-140-3p, miR-26a-5p, miR-29a-3p, miR-30b-5p, miR-338-3p, miR-342-3p, miR-486-5p, miR-652-3p	63.67
miR-140-3p, miR-26a-5p, miR-30b-5p, miR-335-5p, miR-338-3p, miR-342-3p, miR-486-5p, miR-652-3p	63.74
miR-140-3p, miR-26a-5p, miR-30b-5p, miR-338-3p, miR-340-5p, miR-342-3p, miR-486-5p, miR-652-3p	64.28
miR-140-3p, miR-26a-5p, miR-30b-5p, miR-338-3p, miR-342-3p, miR-376a-3p, miR-486-5p, miR-652-3p	61.18
miR-140-3p, miR-181a-5p, miR-22-3p, miR-26a-5p, miR-30b-5p, miR-338-3p, miR-342-3p, miR-486-5p, miR-652-3p	62.88
miR-140-3p, miR-192-5p, miR-22-3p, miR-26a-5p, miR-30b-5p, miR-338-3p, miR-342-3p, miR-486-5p, miR-652-3p	65.02
miR-140-3p, miR-22-3p, miR-26a-5p, miR-29a-3p, miR-30b-5p, miR-338-3p, miR-342-3p, miR-486-5p, miR-652-3p	63.62
miR-140-3p, miR-22-3p, miR-26a-5p, miR-30b-5p, miR-335-5p, miR-338-3p, miR-342-3p, miR-486-5p, miR-652-3p	63.48
miR-140-3p, miR-22-3p, miR-26a-5p, miR-30b-5p, miR-338-3p, miR-340-5p, miR-342-3p, miR-486-5p, miR-652-3p	64.28
miR-140-3p, miR-22-3p, miR-26a-5p, miR-30b-5p, miR-338-3p, miR-342-3p, miR-376a-3p, miR-486-5p, miR-652-3p	63.22
miR-140-3p, miR-181a-5p, miR-192-5p, miR-22-3p, miR-26a-5p, miR-30b-5p, miR-338-3p, miR-342-3p, miR-486-5p, miR-652-3p	64.60
miR-140-3p, miR-192-5p, miR-22-3p, miR-26a-5p, miR-29a-3p, miR-30b-5p, miR-338-3p, miR-342-3p, miR-486-5p, miR-652-3p	65.61
miR-140-3p, miR-192-5p, miR-22-3p, miR-26a-5p, miR-30b-5p, miR-335-5p, miR-338-3p, miR-342-3p, miR-486-5p, miR-652-3p	65.52
miR-140-3p, miR-192-5p, miR-22-3p, miR-26a-5p, miR-30b-5p, miR-338-3p, miR-340-5p, miR-342-3p, miR-486-5p, miR-652-3p	64.31
miR-140-3p, miR-192-5p, miR-22-3p, miR-26a-5p, miR-30b-5p, miR-338-3p, miR-342-3p, miR-376a-3p, miR-486-5p, miR-652-3p	62.88
miR-140-3p, miR-181a-5p, miR-192-5p, miR-22-3p, miR-26a-5p, miR-29a-3p, miR-30b-5p, miR-338-3p, miR-342-3p, miR-486-5p, miR-652-3p	64.37
miR-140-3p, miR-192-5p, miR-22-3p, miR-26a-5p, miR-29a-3p, miR-30b-5p, miR-335-5p, miR-338-3p, miR-342-3p, miR-486-5p, miR-652-3p	64.11
miR-140-3p, miR-192-5p, miR-22-3p, miR-26a-5p, miR-29a-3p, miR-30b-5p, miR-338-3p, miR-340-5p, miR-342-3p, miR-486-5p, miR-652-3p	64.60
miR-140-3p, miR-192-5p, miR-22-3p, miR-26a-5p, miR-29a-3p, miR-30b-5p, miR-338-3p, miR-342-3p, miR-376a-3p, miR-486-5p, miR-652-3p	61.91
miR-140-3p, miR-181a-5p, miR-192-5p, miR-22-3p, miR-26a-5p, miR-29a-3p, miR-30b-5p, miR-338-3p, miR-340-5p, miR-342-3p, miR-486-5p, miR-652-3p	64.66
miR-140-3p, miR-192-5p, miR-22-3p, miR-26a-5p, miR-29a-3p, miR-30b-5p, miR-335-5p, miR-338-3p, miR-340-5p, miR-342-3p, miR-486-5p, miR-652-3p	64.24
miR-140-3p, miR-192-5p, miR-22-3p, miR-26a-5p, miR-29a-3p, miR-30b-5p, miR-338-3p, miR-340-5p, miR-342-3p, miR-376a-3p, miR-486-5p, miR-652-3p	62.05
miR-140-3p, miR-181a-5p, miR-192-5p, miR-22-3p, miR-26a-5p, miR-29a-3p, miR-30b-5p, miR-335-5p, miR-338-3p, miR-340-5p, miR-342-3p, miR-486-5p, miR-652-3p	64.08
miR-140-3p, miR-181a-5p, miR-192-5p, miR-22-3p, miR-26a-5p, miR-29a-3p, miR-30b-5p, miR-338-3p, miR-340-5p, miR-342-3p, miR-376a-3p, miR-486-5p, miR-652-3p	61.57
miR-140-3p, miR-181a-5p, miR-192-5p, miR-22-3p, miR-26a-5p, miR-29a-3p, miR-30b-5p, miR-335-5p, miR-338-3p, miR-340-5p, miR-342-3p, miR-376a-3p, miR-486-5p, miR-652-3p	62.54

The performance assessment of the various models was derived from internal validation, utilizing repeated cross-validation (5 repetitions, 5 folds).

© The Author(s) 2025. Published by Oxford University Press on behalf of European Society of Human Reproduction and Embryology.
This is an Open Access article distributed under the terms of the Creative Commons Attribution License (https://creativecommons.org/licenses/by/4.0/), which permits unrestricted reuse, distribution, and reproduction in any medium, provided the original work is properly cited.
Human Reproduction, 2025, 00, 1–22
https://doi.org/10.1093/humrep/deaf221
Original Article
